# Beyond Tripeptides Two-Step Active Machine Learning for Very Large Data sets

**DOI:** 10.1021/acs.jctc.1c00159

**Published:** 2021-04-27

**Authors:** Alexander van Teijlingen, Tell Tuttle

**Affiliations:** Department of Chemistry, University of Strathclyde, 295 Cathedral Street, Glasgow G1 1XL, U.K.

## Abstract

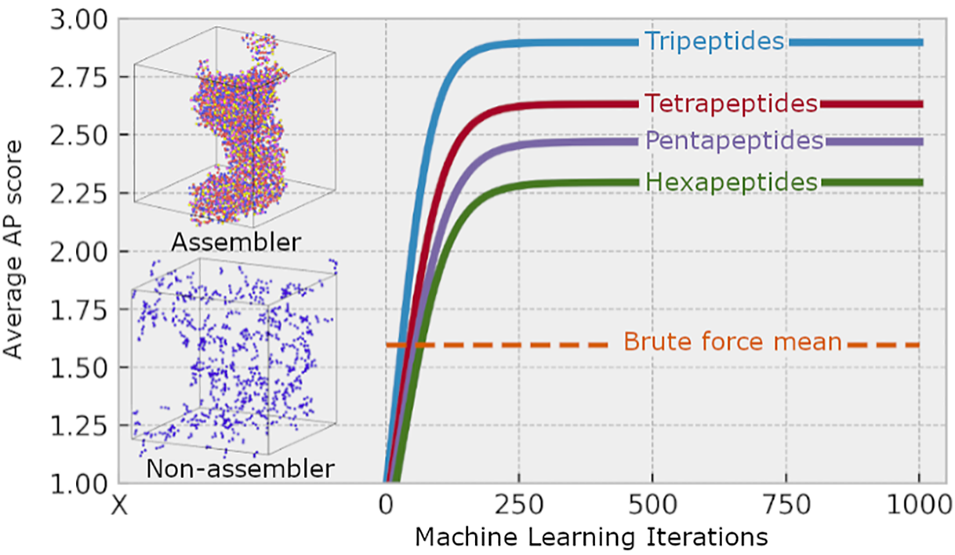

Self-assembling peptide nanostructures have been shown to be of great importance in nature and have presented many promising applications, for example, in medicine as drug-delivery vehicles, biosensors, and antivirals. Being very promising candidates for the growing field of bottom-up manufacture of functional nanomaterials, previous work (Frederix, et al. 2011 and 2015) has screened all possible amino acid combinations for di- and tripeptides in search of such materials. However, the enormous complexity and variety of linear combinations of the 20 amino acids make exhaustive simulation of all combinations of tetrapeptides and above infeasible. Therefore, we have developed an active machine-learning method (also known as “iterative learning” and “evolutionary search method”) which leverages a lower-resolution data set encompassing the whole search space and a just-in-time high-resolution data set which further analyzes those target peptides selected by the lower-resolution model. This model uses newly generated data upon each iteration to improve both lower- and higher-resolution models in the search for ideal candidates. Curation of the lower-resolution data set is explored as a method to control the selected candidates, based on criteria such as log *P*. A major aim of this method is to produce the best results in the least computationally demanding way. This model has been developed to be broadly applicable to other search spaces with minor changes to the algorithm, allowing its use in other areas of research.

## Introduction

Many peptides exhibit the tendency to self-assemble in water into a vast array of different structures, including micelles, nanovesicles, nanotubes, and nanofibers.^1−7^ The inherent biocompatibility of many of these unprotected peptide nanomaterials makes this an attractive class of materials. Recently, there has been a drive in large-scale efforts to identify peptides of interest (antimicrobial,^8−10^ self-assembling,^3,4,11^ antineoplastic,^12−15^*etc.*). This is partially due to the aforementioned biocompatibility, but also to the ease of synthesis which has been automated for short sequences.^16,17^

**Figure 1:**
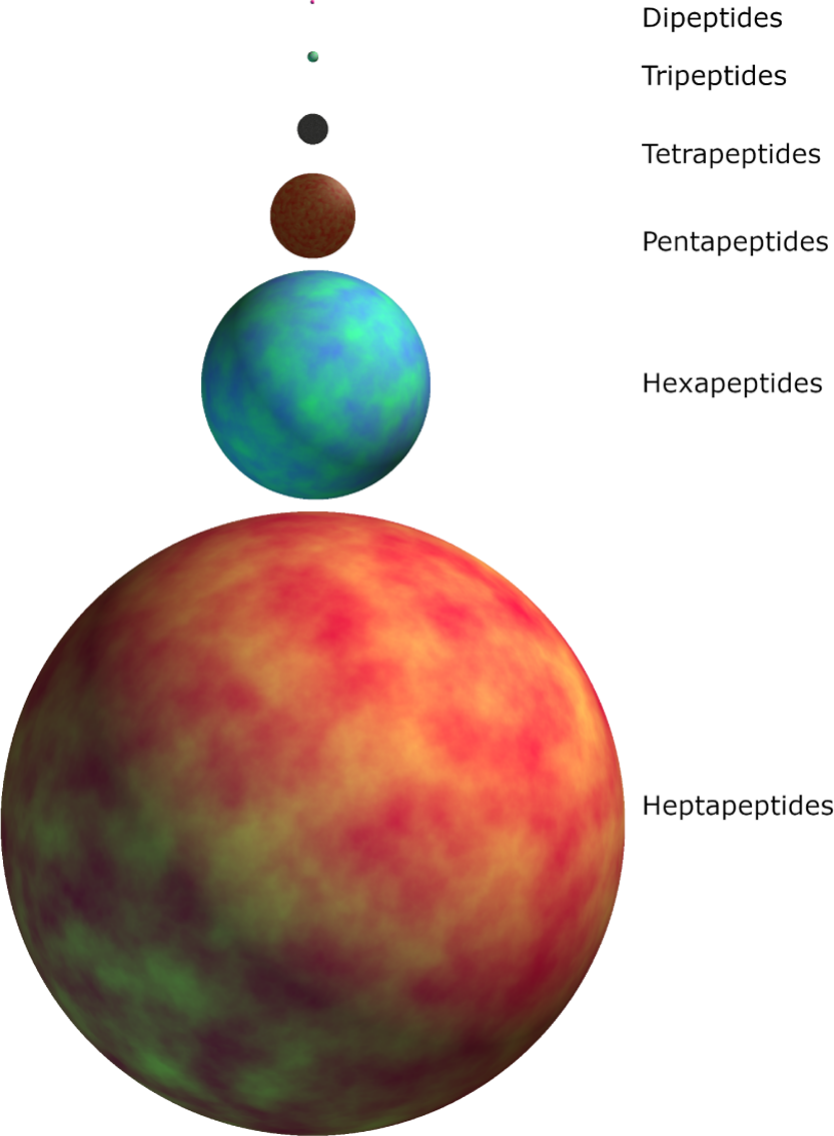
Planetary orbs accurately scaled (by volume) representing the different sizes of different peptide data sets from 2 to 7 amino acid residues.

Despite the ease of synthesis, the discovery of short (di-to octa-) peptides that are able to self-assemble becomes an intractable problem to investigate experimentally due to the vast sequence space that exists for this set of compounds (4 × 10^2^ dipeptides to 2.56 × 10^10^ octapeptides).^18^ However, the use of coarse-grained molecular dynamics (CGMD) simulations to investigate the propensity of the peptides to aggregate (as a precondition of self-assembly) has been employed successfully to guide the selection of candidate peptides for experimental investigation in the case of di- and tripeptides.^3,4^ Unfortunately, the logical next step—a survey of all tetrapeptides—would comprise a search space of 160,000 molecular structures, an achievable but costly (*ca.* 1.6M CPU hours) endeavor, and with pentapeptides, this methodology quickly spins out of control (Figure 1). Our aim is to survey peptides of chain length 4–6 with the intention that this method could be further scaled to peptides of chain length 7–8 with modern computer equipment.

Machine learning is a central focus of the “big data” movement and, as the name suggests, draws conclusions and relationships from large amounts of data. One of the main presumptions of this approach is that as the size of the data increases, the accuracy of the model will also increase. However, if the size of the data set can be increased in a targeted manner (*e.g.,* to include more data points that display the property of interest), then a more efficient approach may be developed. Active learning is a rational sampling method that aims to identify the most informative data to label so that a supervised model trained on this data would perform better than a supervised model trained on an equivalent amount of labeled data chosen at random.^19^ Active learning may also be known as sequential learning as it uses all measures up-to-date to inform the next-best candidate for labeling in an increasingly informed search for the optimal training set with minimal data.^20^

Shmilovich et al. have used this approach to traverse the chemical space of the DXXX-OPV3-XXXD molecular template, where OPV3 represents 1,4-distyrylbenzene and XXX represents variable tripeptides. The search space comprises 8000 (20^3^) molecules as the amino acids on each side are always symmetrical. Their goal was to find the highest performing candidates of this subset of molecules in terms of self-assembly (for further investigation in organo-electronics research) while avoiding performing coarse-grained simulations for each of them. They encode each compound using a variational autoencoder, which projects a high-dimensional molecular representation to a low-dimensional latent space encoding by means of training a neural network to reconstruct these high-dimensional representations after being processed through a bottle-neck layer consisting of fewer nodes than the input/output representation. CGMD simulations are performed in order to quantify aggregation tendency. This information together with the low-dimensional representation is then appended to the training data, and a Gaussian process regressor is retrained and used to predict the next best candidate. This method allowed them to identify a large pool of aggregating peptide derivatives while only running CGMD simulations for 2.3% of the search space, a massive saving in computing resources.^20^

Balachandran et al., in their search for high-temperature ferroelectric perovskites, make use of an out-of-distribution two-step active learning process. They confine their search to perovskite structures of the formula *x*Bi[Me′*y*Me^″^(1 – *y*)]O_3_ – (1 – *x*)PbTiO_3_, perovskites targeting those with high ferroelectric Curie temperature (*T*_c_), and begin training from an initial data set of 167 polycrystalline ceramic samples from the literature. In the first step, a binary classifying support vector machine which uses a radial basis function kernel (SVM_RBF_) is trained to identify highly (>95%) phase-pure perovskite structures. From the identified candidates, a regressor SVM_RBF_ model is trained to predict *T*_c_ of perovskite structures. This two-step model yields a structure with two desirable features and reduces the amount of computational power needed as opposed to simultaneously predicting both features. The best candidate identified from this process is then synthesized, characterized, and the data were added back into the training set using a human-in-the-loop; the process is then repeated. Out of the 10 cycles of this process, the best overall candidate was not predicted until the 10th iteration, which lends weight to the validity of this being an active learning process.^21^

Herein is described and validated a method of using machine-learning models to aid in the search of vast amino acid-combinatorial search space for self-assembling peptides. To achieve this, an iterative machine learning process, often referred to as “active learning”, was implemented which utilizes two SVM_RBF_ regressor models to filter the data set in a two-step process for selecting the candidates for CGMD. This method is demonstrated to be able to select aggregating soluble peptides.

Our method assumes that an out-of-distribution active learning process will be able to extrapolate to peptides with a greater ability to aggregate than existing within the training set. In the literature, out-of-distribution predictions have been shown to work well in the field of computational chemistry. In particular, Sparks et al. demonstrate this by splitting a data set of crystal thermal expansion into training (bottom 85%) and testing/“extraordinary” sets. The study demonstrates that, with high precision and recall, ridge and logistic models are both able to predict which materials are within the “extraordinary” category (defined as within the top 3, 7, 11, and 15%) with a precision-recall area under the curve (AUC) of 0.7–0.8 in all cases.^22^ Such examples of out-of-distribution learning fortify our efforts in implementing an out-of-distribution active learning method for finding self-assembling peptides.

## Methodology

### Computational Methods

Simulations of each peptide were set up using the GROMACS^23^ software package; this program was also used to measure solvent accessible surface area (SASA) of the peptide at initial (*t* = 0 ns) and final (*t* = 200 ns) configurations. The Judred parameters were generated for each peptide using a script written in the Julia programming language;^24^ all other programming was done using the Python programming language.^25^ Mordred parameters were generated using the original Mordred program,^26^ while implementations of the machine-learning algorithms were accessed *via* the scikit-learn (sklearn, version 0.21.3) Python module.^27^ Visualizations of molecular ensembles were rendered and displayed by the software package OVITO.^28^ The SASA was used to determine the AP score; by measuring this value at the beginning and end of the molecular dynamics simulations, the AP can be calculated according to eq 1. The utility of the AP score has already been shown to be a useful measure to predict self-assembly;^3,4^ as such in the current work, we extend the already existing predictive ability of this measure to larger peptide sets than could previously be studied.
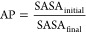
1

The parameters for coarse-grained peptide, water, and ion molecules are those of the MARTINI forcefield (version 2.2).^29^ The peptide atoms are mapped one-to-four in corresponding atoms-to-beads, the water beads represent four water molecules for the purpose of computational efficiency and the ion beads represent one ion atom. This causes an inevitable loss of detail (such as hydrogen bonding, specific atom–atom interactions, *etc.*) but leaves a much more computationally efficient method of studying aggregation as the atom property descriptions required (polarity, molecular shape, bond lengths, *etc.*) are implied *via* the coarse-grained representation. This advantage was increasingly warranted as the magnitude of molecules understudy increases. Each cubic *NPT* box was filled by randomly placing 300 zwitterionic peptides with a minimum of 0.3 nm inter-molecular distance in a pre-equilibrated 12.5 nm^3^ MARTINI water box such that the final concentration was *ca.* 0.4 M. The temperature and pressure were kept constant at 303 K and 1 bar, respectively, *via* a v-rescale and Berendsen barostat.^30,31^ Bond lengths between backbone and side-chain for peptides I, V, and Y as well as aromatic side chains were constrained *via* the LINCS algorithm.^32^ The boxes were minimized using the steepest descent integrator and equilibrated for 200 ns. Due to the relationship between the diffusion constants of the MARTINI coarse-grained and atomistic simulations, the effective simulation time is four times greater than the formal simulation time. Herein, we refer to the effective simulation time and not the formal time.

### Molecular Descriptors

**Figure 2:**
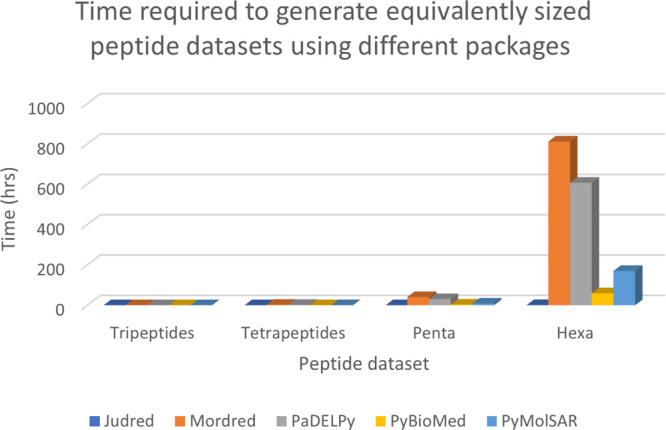
Approximate amount of time required to generate the equivalent data sets explored in this study (tri-to hexapeptides) using different descriptor generating packages. Scaling further, for example, to octapeptides would still be realistically for the Judred descriptors, requiring 12 CPU days while even for the next faster descriptor generator would require three CPU years.

This method for discovery of self-assembling water-soluble peptides employs a two-step active learning process. Initially, Mordred^26^ descriptors were used as the sole representative of the peptides as they provided useful and interpretable data to the machine-learning algorithm that allowed the prediction of AP scores with low error (Table 3). However, it was found that these data were too resource-intensive to generate for the larger data sets (Figure 2) as they require the use of RDKit^33^ mol data structures which are relatively resource-intensive processes to generate and query compared to the table lookup method we propose. This feature of using RDKit, Open Babel,^34^ the Chemistry Development Kit,^35^ or other representations to calculate descriptors for peptides is very common and is present in a number of packages including PyBioMed,^36^ chemdescriptor,^37^ PaDELPy,^38^ and PyMolSAR.^39^ While these representations have the obvious benefit of being able to calculate more descriptors that more closely model the real structure, they are significantly more resource-intensive. As such, where an accurate model can be built without them is prudent to do so, particularly when attempting to search very large sequence spaces.

The original Mordred parameter set (1231 parameters) was reduced to 47 parameters (see Table S1, Supporting Information) *via* 5-fold recursive feature elimination across the initial samples for three data sets (tetra-to hexapeptides). This reduction was found to improve prediction of the AP scores for all data sets (*vida infra*). Two correlation matrices for the selected Mordred features have been visualized in Supporting Information (Figures S1 and S2). This increased accuracy is most likely due to reduced noise in the data but reducing the number of features also has the added benefit of allowing for faster predictions and optimizing of hyperparameters, an important consideration for many machine-learning models that do not scale well with increasing numbers of features.

Reducing the number of features is not sufficient to overcome the time requirements of exhaustively generating Mordred (or other molecular representation) parameters for all peptides of large data sets. Thus, a simplified Judred (a concatenation of “Julia”, the programming language, and Mordred, the descriptor generator) software package was created to address these problems. The Judred program generates 10 parameters (Table 1) which were chosen based on two constraints: (1) only the one-letter peptide codes are required to calculate the parameters. By looking up and processing values from a table as opposed to generating molecular structures, the Judred approach provides a low-resolution screening that is applicable to massive search spaces (Figure 1). (2) The parameters should act as a proxy for the type of intermolecular forces that drive self-assembly (*e.g.,* the number of SP2 atoms in a peptide is an indication of the rigidity of the system and thus the role of entropic loss upon forming a self-assembled structure, Table 1). The nature of the Judred parameters allows for search space scaling >4 orders of magnitude greater than any other attempt at using machine learning or other means to search peptide self-assembly space. The implementation has been made available on GitHub.

**Table 1:** 10 Parameters Generated by the Judred Program for Use in Pre-screening of Data sets *via* Random Forest Regression^a^

name	description	physical mechanism
SP2	number of SP2 carbon atoms	entropic loss
NH2	number of NH2/NH3 groups on the side chain(s)	hydrogen bonding
MW	molecular weight	size
S	number of sulfur atoms	hydrogen bonding
log *P* WW	Wimley–White log *P*^40,41^	solubility
*Z*	charge	electrostatic interactions
RotRatio	ratio of SP2 to SP3 carbon atoms	relative entropic loss
MaxASA	maximum solvent accessible surface^42^	hydrophobic effect
Bulkiness	sum of amino acid bulkiness^43^	size
OH	number of OH groups (excluding backbone)	hydrogen bonding

a Listed is a brief description of each parameter as well as the physical mechanisms it is approximating.

### Model Selection

The input data for tripeptides and AP scores were split into a training (80%) and test set (20%). For each machine learning model listed in Table 2, a wide range of hyperparameters were optimized *via* fivefold cross-validation of the training set and accuracy in predicting the test set was compared. It was found that for the Judred data, the gradient boosting regressor (GBR), decision tree, SVM_RBF_, and multi-layer perception (MLP) performed equally well, while for the Mordred data, the SVM_RBF_ and MLP were the best predictors. However, in both cases, as the Matthews correlation coefficient (MCC) suggest the SVM_RBF_ to be better at classifying high-scoring AP peptides from lower scoring ones, this model was chosen. The optimal hyperparameters for both the Judred and Mordred SVM_RBF_ models were found to be very similar and for the sake of aspiring to least complexity, we will use the SVM_RBF_ model for both steps of the algorithm with the same set of hyperparameters (hyperparameters for each model can be found in Supporting Information, Section 1.5).

**Table 2:** Metrics for Different Machine Learning Algorithms Using the Judred and Mordred Data sets Comparatively^a^

	MSE	MAE	*R*^2^	MCC	AUC
Judred					
SVM_RBF_	0.0152	0.0941	0.93	0.88	0.99
linear SVM	0.0529	0.1884	0.75	0.69	0.96
gradient boosting regressor	0.0146	0.0890	0.93	0.87	0.99
elastic net	0.0533	0.1875	0.75	0.70	0.96
random forest	0.0478	0.1699	0.78	0.79	0.97
ridge	0.0527	0.1864	0.75	0.70	0.96
multi-layer perceptron	0.0144	0.0892	0.93	0.86	0.99
stochastic gradient descent	0.0513	0.1835	0.76	0.70	0.96
decision tree	0.0150	0.0899	0.93	0.86	0.99
Mordred					
SVM_RBF_	0.0072	0.0673	0.97	0.92	0.99
linear SVM	0.0232	0.1225	0.89	0.82	0.98
gradient boosting regressor	0.0088	0.0708	0.96	0.88	0.99
elastic net	0.0268	0.1310	0.87	0.78	0.98
random forest	0.0475	0.1693	0.78	0.73	0.97
ridge	0.0242	0.1244	0.87	0.82	0.98
multi-layer perceptron	0.0065	0.0609	0.97	0.90	0.99
stochastic gradient descent	0.0248	0.1270	0.88	0.82	0.99
decision tree	0.0177	0.0933	0.92	0.85	0.99

a An 80–20 train-test split was used to determine the best models according to five metrics; for MCC, a cut-off of AP = 2.0 was used. Receiver operating characteristic curves as well as testing on a 66–34 train-test split are included in the Supporting Information, Figure S3 and Table S3.

### Initial Training Set

We found that the model was able to learn to correctly identify high AP scoring peptides without the curation of any “good” initial training set; therefore, we only include a single peptide in the initial training set; for this, we selected polyalanine of relevant chain length with respect to the data set. The first iteration returns a random set of 10 peptides as it is not possible to train a regressor with a single peptide; however, this is often sufficient to immediately begin finding high AP peptides from the second iteration with continuing improvement of the model allowing the model to rapidly learn patterns that result in high AP scores. In order to discern if the model was being biased heavily by this initial peptide, we run the model 20 times each time starting with a different sequence-uniform tripeptide; we found the maximum difference between any two mean AP values after 10 iterations to be 0.078, falling to 0.074 after 20 iterations; the plots have been visualized in the Supporting Information, Figure S4.

### Active Learning

After selecting the initial training sample, an SVM_RBF_ regressor was used to predict AP scores from the Judred data and return the top *N* potential peptides to the algorithm depending on the length of the peptide chains (eq 2) which acts as an indicator of data set size. This is to provide some way of scaling the algorithm without causing runaway second-step-screening sizes that would significantly hinder the speed of the algorithm.
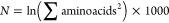
2

**Figure 3:**
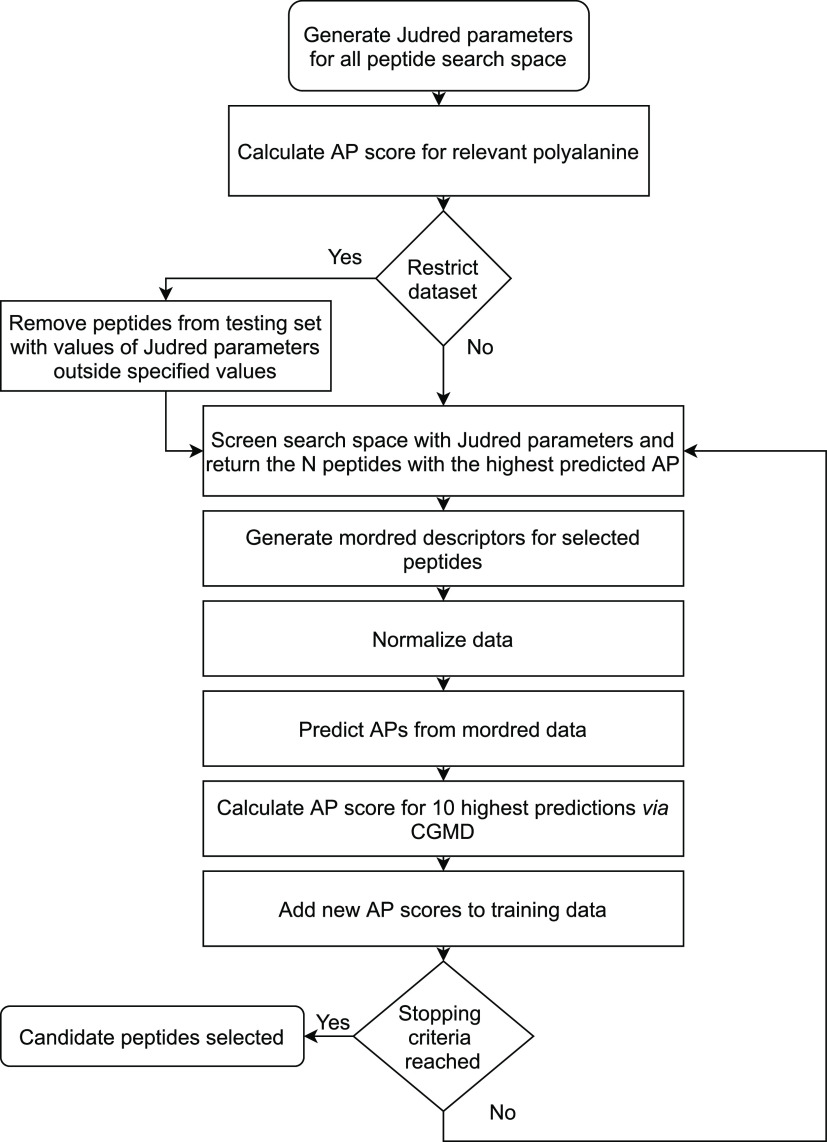
Flow chart of the process developed in this study to find peptides that aggregate in water *via* an active learning method. The stopping criteria in our experiments was a limit on the number of iterations.

From these *N* peptides, the Mordred descriptors are generated and an SVM_RBF_ model predicts the APs using those descriptors, the top 10 peptides from these are then submitted for CGMD simulations, the APs from these simulations are measured, and these values are added to the training set of both the Judred and Mordred regressors. The pre-screening subroutine returns a much larger number of peptides than will be submitted for CGMD simulation as a precaution against losing potentially interesting peptides from the lower accuracy pre-screening model while retaining a reasonable run time such that the CGMD simulations remain the rate-limiting step. The loop will terminate on a given criterion for which this example uses a limit to the number of iterations allowed but allows for result-specific criteria such as a peptide with an AP above a certain value. The complete active learning algorithm is depicted in Figure 3.

Data sets may be further pre-processed by removing peptides outside of defined ranges of Judred parameters. For example, prior to training machine-learning models, insoluble peptides (log *P* > 0) were removed from the data set according to the peptide specific log *P* (eq S1, Supporting Information) proposed by Wimley and White^40,41^ so that our search was restricted to water-soluble aggregating peptides. This process can be performed for any parameter or combination of Judred parameters, for example, restricting search space of peptides with a positive charge, or a charge of −2 only or peptides that have a charge of +2 and a molecular weight of <100 amu. This approach has been applied in this work to test different data sets for soluble peptides, peptide with log *P* < 0 and log *P* < −4.

## Results and Discussion

### Aggregation Propensity as a Target Property

**Figure 4:**
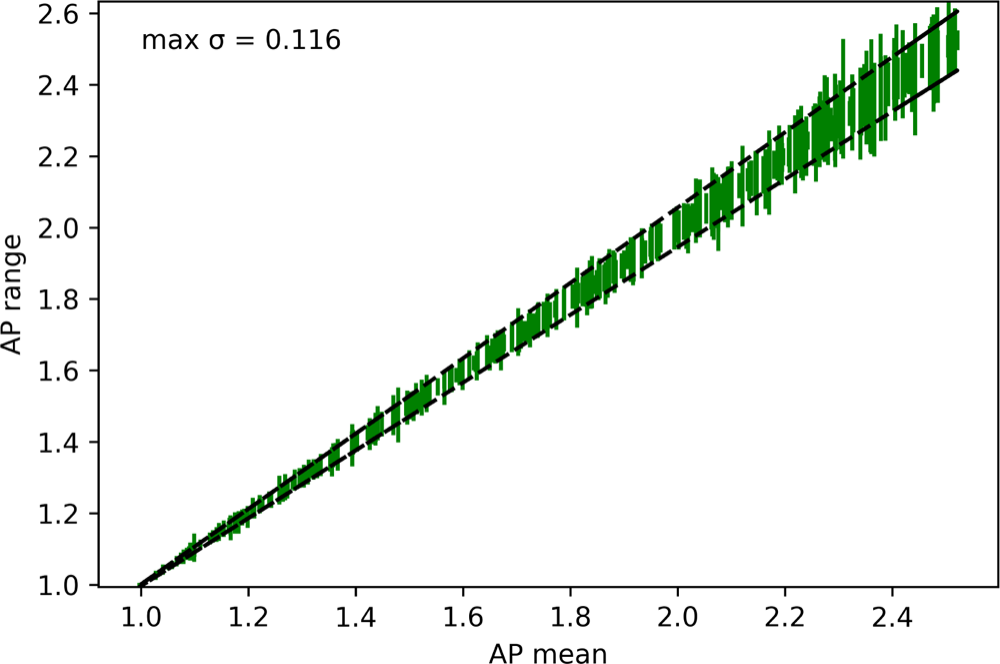
As the AP score increases, the range of values produced by separate CGMD simulations increase. 80 peptides within the range of AP = 1.0–2.6 were each selected for data sets tetra-to hexapeptides by randomly selecting from lists to achieve an approximately even distribution, across both size and AP. The two trend lines shown are the linear best fit between the mean AP and the max/min in each set.

The active learning method developed in this work is focused on predicting the AP score for a peptide and as such is trained on the AP scores resulting from CGMD simulations. Therefore, the robustness of this score is critical. To investigate this, five repeat simulations of 80 peptides of AP ranges 1–2.6 of peptides from the tetra-, penta-, and hexapeptides were carried out to determine the experimental error (Figure 4).

The standard deviation in the AP scores tends to increase with increasing AP, due to the different ways and orientations that peptides may aggregate and varies with starting positions and velocities (Figure 4). Nonetheless, the standard deviation remains low in all cases, confirming the robustness of the AP score as a measure for the ability of the peptides to aggregate. Furthermore, the results show that the dependence on the standard deviation on the AP score is the same between peptides of different chain lengths. In all tested sequences, the maximum standard deviation was less than 0.12.

**Figure 5:**
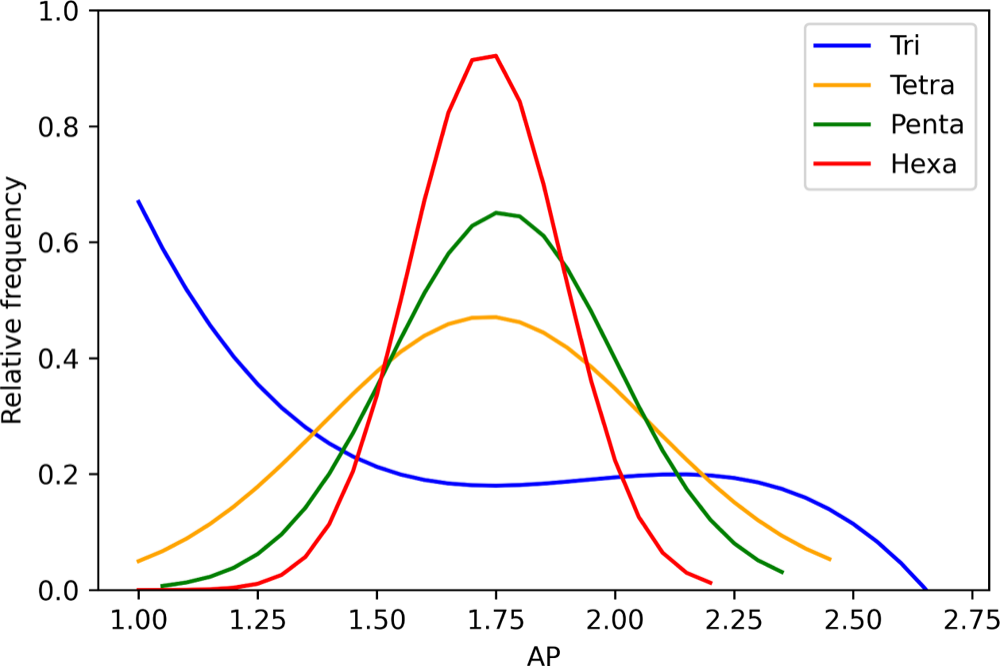
Range of APs from random sets of 800 peptides from varying peptide length data sets; the tripeptide distribution fits very well to a 3rd order polynomial, while the tetra–hexapeptide data sets fit much more closely to Gaussian distributions.

**Figure 6:**
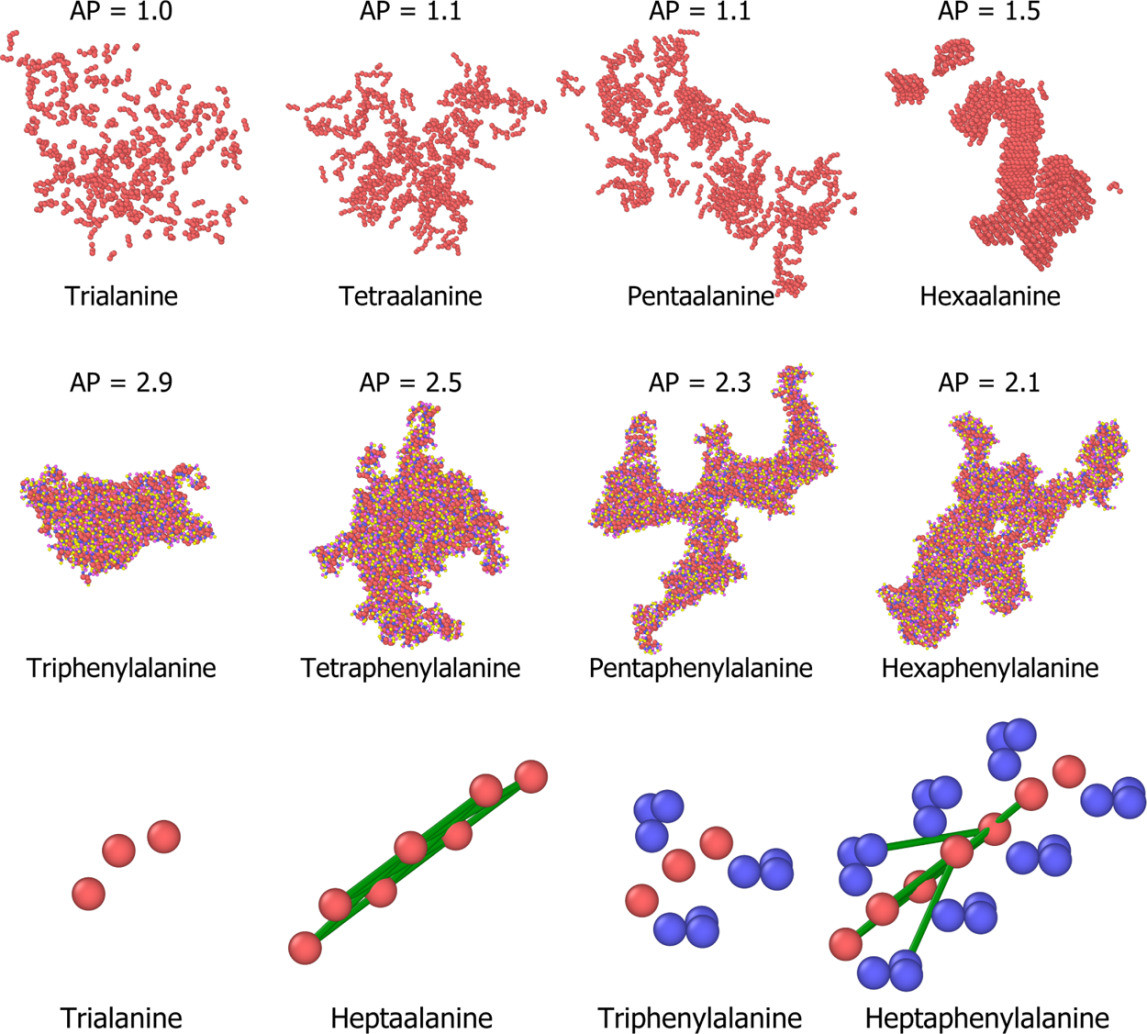
Narrowing of AP ranges in tri–hexapeptide data sets is exemplified by the increasing AP of polyalanine with chain length and decreasing AP for polyphenylalanine with increasing chain length rows 1 and 2. This is due in part to the way the MARTINI force field constructs weak long-range elastic bonds for peptides of chain length 4 or more, visualized in green in the bottom row.

We found the mean variance in the AP score calculated at the final nine frames (196–200 ns) to be <0.0002 with a maximum value of 0.0019, and therefore, it is sufficient to only use initial and the final frame to calculate AP rather than any average of frames. We also found that the range of AP scores tended to decrease with increasing peptide chain length; this has been visualized in Figure 5 from sets of 800 randomly selected peptides in each data set. Figure 6 also shows two examples of changing AP with peptide chain length; the first is the increase in AP for polyalanine, particularly due to beta-sheet formation at the hexapeptide stage and the decrease in AP for polyphenylalanine as the structure begins to become more branched and therefore has more surface area; the narrowing in range is also in-part due to the to construction of weak long-range elastic bonds for peptide of chain length ≥4 in the MARTINI force field which reduce SASA_initial_ per amino acid, visualized in green at the bottom of Figure 6.

### Performance of Judred and Mordred Models

**Figure 7:**
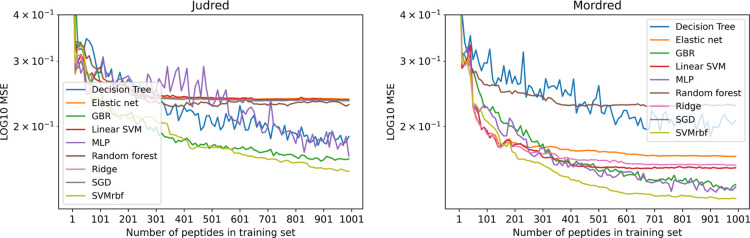
Models that are able to learn non-linear relationships show the greatest increase in accuracy as the size of the training set increases; of these, the SVM_RBF_ showed the best accuracy improvement trend and lowest final MSE.

The ability of the newly defined Judred parameters and the much larger set of Mordred parameters to predict the AP score of unknown peptides was benchmarked against the known AP scores of the tripeptide series. For this purpose, the MSE between the known and predicted AP scores determined by the different ML algorithms was investigated and the MSE was measured as a function of training set size for each model and both parameter sets (Judred and Mordred, Figure 7). We find in both instances that the SVM_RBF_ showed the best accuracy improvement trend, finishing at the lowest MSE and decreasing rapidly with the training set size.

In addition to the analysis based on the known tripeptide AP scores, the accuracy of the Judred and Mordred models was measured *via* fivefold CV against 800 randomly selected peptides from each of the peptide data sets (tri–hexapeptides, Table 3). The error in prediction decreases as the data set size increases, while the *R*^2^ score decreases; this is due to the narrowing of the AP score range as the peptide chain length increases (Figure 5). The model predictions for each data set have been plotted in the Supporting Information, Figure S5.

**Table 3:** For Each Set of Peptides, the Accuracy of the SVM_RBF_ Models with Judred or Mordred Parameters is Measured Across Each Peptide Chain Length (Tri–Hexapeptides) *via* fivefold Cross-Validation of 800 Randomly Selected Peptides

	Judred			Mordred		
	*R*^2^	RMSE	MAE	*R*^2^	RMSE	MAE
hexa	0.85	0.0776	0.0621	0.87	0.0704	0.0565
penta	0.86	0.0939	0.0760	0.89	0.082	0.0658
tetra	0.87	0.1155	0.0899	0.88	0.1092	0.0859
tri	0.89	0.1515	0.1135	0.91	0.1342	0.1064

The error was found to be greater in all instances for all data sets for the Judred model over the Mordred model; this is to be expected from a data set containing less parameters; the lower accuracy of the Judred model is compensated by the relative speed of generating the data set (Figure 2) and its use as a pre-screening method.

### Active Learning *Versus* Screening

**Figure 8:**
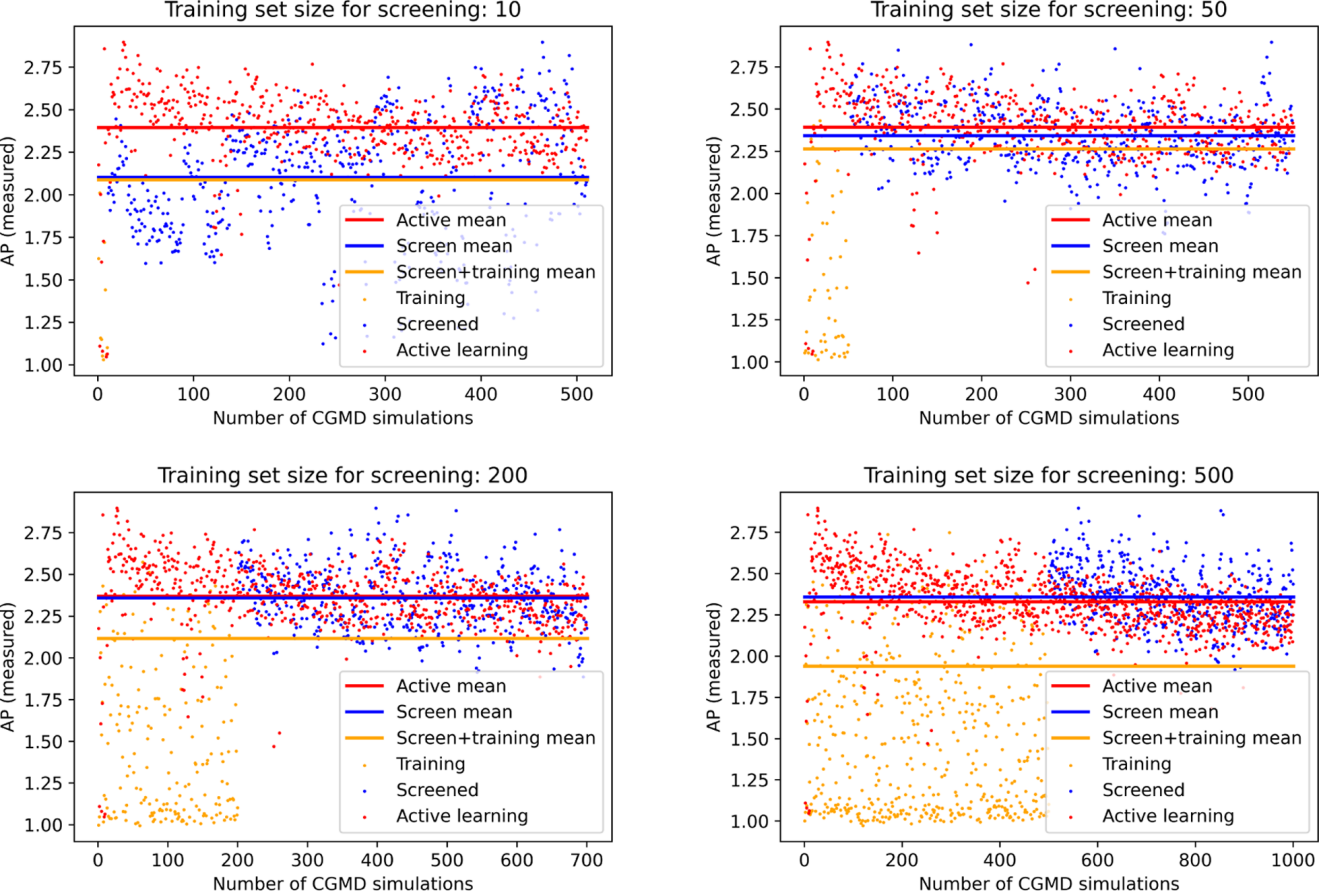
Comparison of active learning *versus* screening for tripeptides; in each case, the active learning algorithm begins with trialanine and learns to predict high AP peptides, while the screening method is provided with a training set of increasing size. While the training size of 500 peptides yields a slightly higher average AP score for the screened peptides (blue) than the active learning algorithm (red), when taking into account the total number of CGMD simulations (orange), which include the training set, the screening method is unable to outperform the active learning method.

It is important when comparing active learning to screening to include the training set, as these CGMD simulations are unavoidable and distort the comparison in the favor of screening due to active learning blurring the lines between training and selected peptides. For example, a screening process to predict 100 peptides from a 1000 member training set should take its average AP from all 1100 CGMD simulations that had to be run in order to obtain the top 100. We have visualized what this looks like in Figure 8, where the training set size for the screening model contains only 10 members and we see a clear performance boost for the active learning model which is to be expected as each iteration adds to the active learning training set, that is, by the 49th iteration (CGMD simulations 290–300), there is a training set size of 490. This advantage appears to be lost when the training set size for the screening model has increased to 500 members (blue line *vs* red line); however, the actual number of CGMD simulations is not the 500 screened but the 1000 in total, which falls short of the active learning process (orange line *vs* red line).

### Active Learning with Data Set Restrictions

This method was first validated by using the model to predict the top performing tripeptides identified by Tuttle et al.^3^ both in the AP (aggregating) and AP_H_ (aggregating and soluble) categories. Each of these categories contains a reported list of 20 self-assembling tripeptides with a total number of 39 unique tripeptides (PFF appears in both lists). Our model managed to find 35 of the 39 unique tripeptides and 20/20 of the purely high AP peptides (Table 4), demonstrating the model’s ability to find the top self-assembling peptides without having to simulate, or even generate high-resolution descriptors for, peptides.

**Table 4:** Iteration of Different Tripeptides Where Found When Running the Model with Different Data Set Restrictions^a^

iteration	tripeptides no restrict	tripeptides log *P* < 0	tripeptides log *P* < 3
1	FFF		
2	WFF, FWF, FFW		
3	FWI		
4			KWD, KHD, WKD
5	IFW,FYI, PWF		HKD, KYD, KFD
6	WFL	SSF	KWE
7			WKE, KEH
8			KYE
9	IFF, PFF		KHE
10	FFM		
11	WFF, VFW		
12	VFF		
13		SCW	
14	MFF		
≥15	WLL,SFW, IMW, LCF	KWF, KFW	
not found:	PCF, TSF, GFF, VAW		

a 35/39 of the unique top performing tripeptides reported by Tuttle et al.^3^ were found by our method within 15 iterations. All the high AP peptides are in the left column, and all the peptides in the restricted data sets are from the high AP_H_ collection.

**Figure 9:**
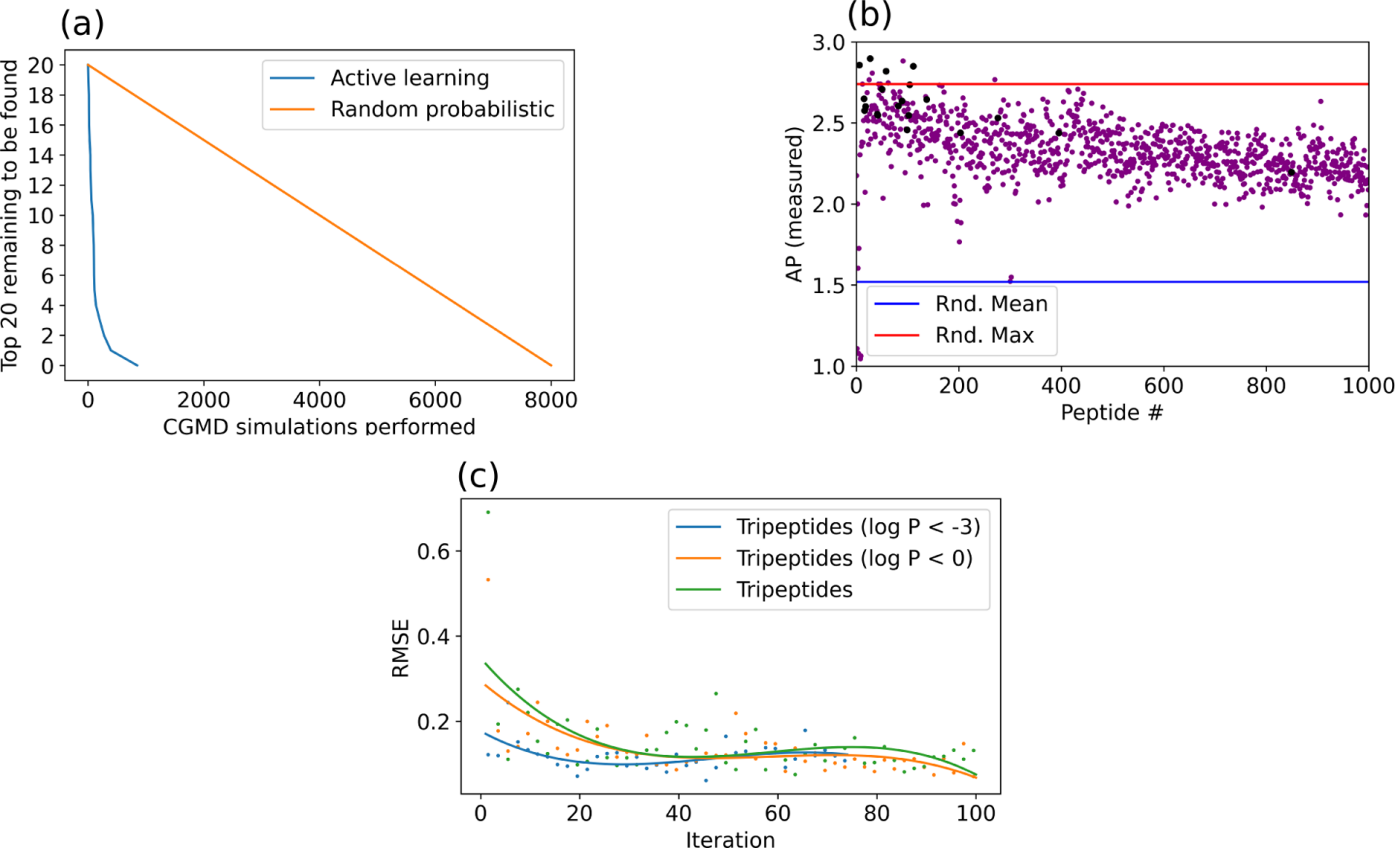
(a) Number of top 20 tripeptides reported by Tuttle et al.^3^ found *via* active learning compared to the number of CGMD simulations performed *versus* how they would likely be discovered *via* whole data set screening. (b) AP of each peptide found *via* the active learning process; those reported by Tuttle et al.^3^ are shown in black, with the mean and maximum values from a set of 800 randomly selected tripeptides. (c) Polynomial best-fit of RMSE AP predictions of the peptides selected in the next iteration of active learning *versus* iteration.

The ability of the model to identify the best candidates within 15 iterations is an indication of the power of this approach. The 15 iterations of the model implies that a maximum of 150 CGMD simulations were carried out to achieve these results. In contrast, the initial tripeptide study required 8000 CGMD simulations to map the complete search space.^3^ Given that the CGMD simulations are the rate-limiting step in the active learning process, this leads to a >50× acceleration in the discovery process. This dramatic reduction in the number of CGMD simulations required has been visualized in Figure 9 along with improvements in predictions over iterations of active learning. We compared this method of iterating with the top 10 best predictions with an alternative method, where we iterate with the top peptide and nine randomly selected weighted tripeptides (where each weight for random sampling is equal to AP); this alternative method found 19 of the top 20 tripeptides with an overall mean of 2.21; by contrast, the method implemented herein whereby only the top predictions are simulated produced a mean AP of 2.33; the results are given in Figure S6, Supporting Information. We also compare our model to a Judred-only version of the model (Figure S7, Supporting Information) and find that the Judred-only version tends to learn slower and produce more erratic results.

**Figure 10:**
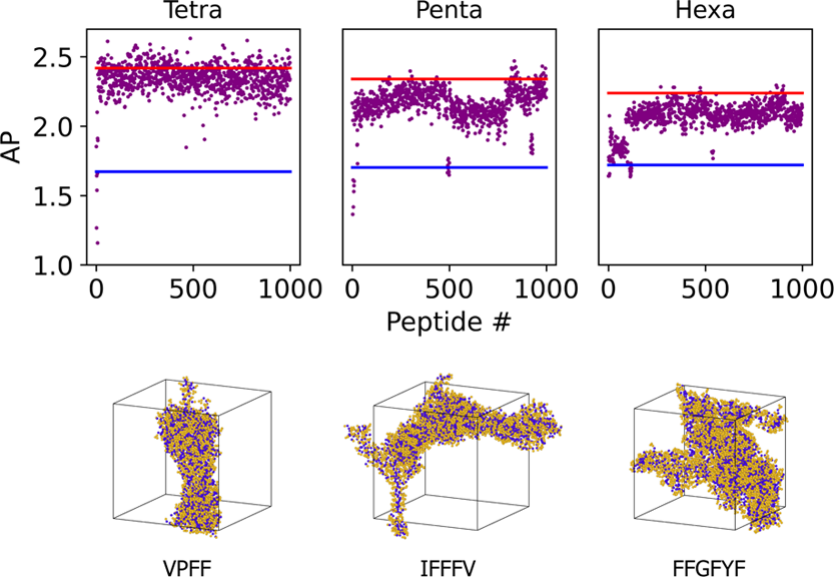
Algorithm learns to predict high AP peptides rapidly and continues to find peptides above the maximum and mean AP in the random set of 800 (red and blue lines respectively) over 100 iterations. The first iteration did not provide enough data to allow the algorithm to predict above random maximum in the second iteration (penta- and hexapeptides); the subsequent iterations show continued improvement and even self-correction (pentapeptides) where the predictions began to slide. The speed of the algorithm to learn to predict the top performing peptides is dependent on the nature of the initial iteration of random peptides and not related to the size of the data set.

In the application of the active learning method to large (peptide length 4–6) unrestricted data sets, each model was run for 100 iterations selecting 10 peptides from each. The first test examined an unrestricted data set of each search. This revealed that the result, while true to the target conditions, consisted of >99% insoluble peptides (Figure 10). This suggests that the aggregation is related to (but not necessarily limited by) the peptide solubility rather than specific intermolecular interactions, which are required for the aggregation to progress to an ordered (self-assembled) state.

**Figure 11:**
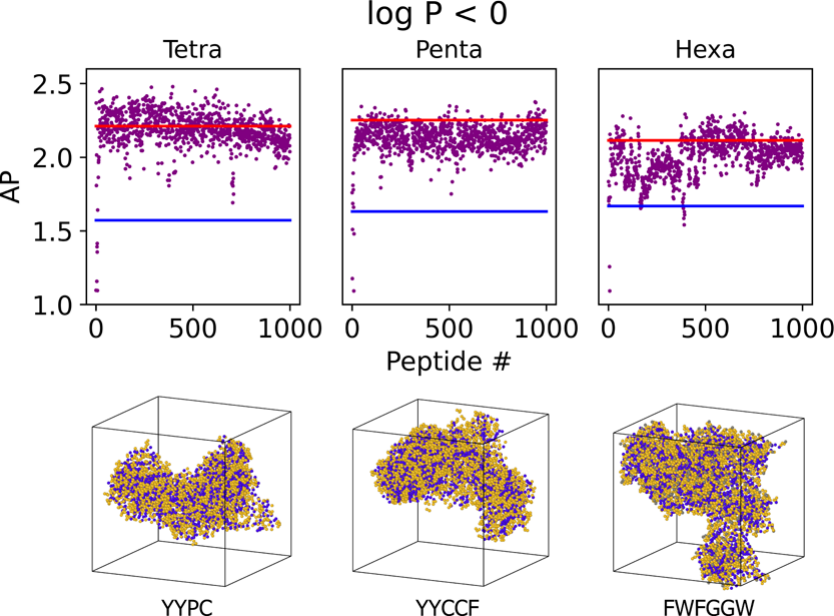
Self-assembling soluble (log *P* < 0) peptides with high AP score found *via* active learning. In each case, the model finds peptides above the maximum and mean values from the random set (red and blue lines respectively). Each of the three models show upward trends demonstrating the active learning process. In the case of: tetrapeptides, the highest scoring peptide was found at number 395 (YYPC); pentapeptides, the highest scoring peptide was found at number 797 (YYCCF); hexapeptides, the highest scoring peptide was found at number 702 (FWFGGW). Snapshots of the highest scoring peptides are shown below each graph at *t* = 200 ns. Purple beads represent peptide backbones and orange beads represent the side-chains. Water molecules have been omitted from the visualization, and the peptide periodic boundary conditions unwrapped at the box edges *via* clustering and centring. Peptides found *via* active learning that have been previously reported in the literature are given at the end (Table 5).

To search for those peptides that have a higher chance of self-assembling, rather than simply aggregating and precipitating out of the solution, this model was used to find water-soluble (log *P* < 0) aggregating peptides to validate the methodology’s ability to find self-assembling peptides. It was found that the AP scores for the actively selected peptides tend to increase over time (Figure 11), where the first iteration did not provide the model with sufficient information; this indicates that the model is self-improving. In each case, the model was able to learn to predict peptides with AP scores above the random maximum and >99% peptides predicted were above the random mean.

**Figure 12:**
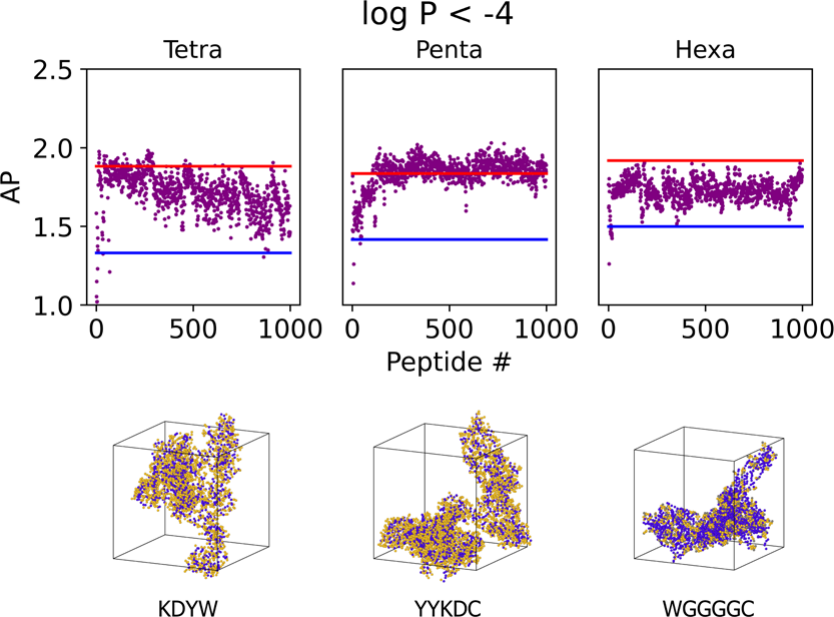
Self-assembling peptides with high AP score with log *P* < −4 found *via* active learning; in each case, the models find peptides above the maximum and mean values from the random set (red and blue lines, respectively). The trend seems to be at a restriction of this magnitude; the larger the data set, the better the model is able to learn.

These restrictions (log *P* < 0) allow the algorithm to find soluble peptides that aggregate in water. However, amino acid analysis of these peptides shows a heavy reliance on selecting for aromatic (Phe/Tyr/Trp) sequences which are well known to promote aggregation. Increasing the restriction on log *P* to only include peptides of log *P* < −4 showed that this still tended to select for aromatic moieties with charged moieties within the peptide (Figure 12a). This restriction found on average peptides with lower AP scores (though still aggregating) than with the log *P* < 0 restriction. Hydrophilic self-assembling peptides are excellent candidates for hydrogels and can serve as potential drug-delivery vehicles;^44,45^ thus, our method of iteratively searching for and measuring aggregation of controllable (by modifying the log *P* requirement) hydrophilic peptides may prove useful in this field, particularly in the directly delivery of hydrophobic antineoplastic molecules.^44^

A non-exhaustive manual search of the literature was performed to find tetrapeptides reported by the active learning model reported herein. In doing so, we back-validate this process against experimental data in order to demonstrate that the other aggregating peptides found *via* this method are likely to be validated experimentally. For each data set, the list of peptides that have been found in the literature have been reported in Table 5.

**Table 5:** Self-Assembling or Aggregating Peptides Found by the Active Learning Algorithm Described Herein has Found a Number That Has Already Been Reported in the Literature, Either Explicitly as Aggregating or Self-Assembling or Implicated as an Important Motif in the Self-Assembly or Aggregation of a Large Peptide/Protein

tetra1000	iteration	citation
FFFA	12	Liang et al.^46^
FFFF	12	Mayans et al.^47^
FFPP	16	Joshi and Verma^48^
FPFP	29	Joshi and Verma^48^
FPPF	30	Joshi and Verma^48^
YPFF	48	Szegedi et al.^49^
LVFF	72	Lampel et al.^50^
VFFA	99	Liang et al.^46^

tetra0	iteration	citation
CYFC	19	Taioli et al.^51^
YPGY	90	Lemire et al.^52^
YWCS	94	Shekhar and Dey^53^

tetra-4	iteration	citation
RGDH	2	Mei et al.^54^
DRGH	9	Moon et al.^55^
DSYG	23	Becker et al.^56^
YRGD	94	Garagorri et al.^57^

## Conclusions

The main challenge of exploring combinatorial space is the explosion of possible sequences that enumerate from all possible combinations. Past work has shown that with modern processing capability, all di- (400) and tripeptides (8000) may be simulated *via* molecular dynamics. However, this is infeasible for tetrapeptides and impossible, for now, for hexapeptides and beyond. The model presented in this work has successfully identified 35 of the top 39 tripeptides (20/20 of the top AP scores), demonstrating a >50× acceleration over whole-search space screening methods. However, it has also shown how even through the use of machine-learning algorithms, generating enough descriptors for the much larger peptide data sets can prove overbearing. Therefore, we have used a two-step machine learning model capable of pre-screening extremely large data sets *via* the use of lower precision molecular descriptors providing a specifically narrower view of the data set for further investigation by a higher precision model.

The active learning model we have presented herein traversed tens of millions of peptides in search of candidates with a desirable property (high AP). Moreover, the general approach presented may be extended to larger compound classes (octapeptides and proteins) or different compound classes (peptoids, lipids, metal–organic frameworks, *etc.*). Our model leverages the relative advantages of low- and high-resolution data sets to search for molecules of interest based on a specified criterion while iteratively improving its own searching capacity (*via* addition of new data closer to the target property). The low-resolution (Judred) search allows that the entirety of the search space is traversed and selects a long list of potential candidates for the more computationally expensive high-resolution (Mordred) screening which in turns selected the candidate peptides for molecular dynamics simulation. This allows for both the outcome of the simulation and even the high-resolution descriptors to be unknown at the beginning of the search.

## Supporting Information Available

Computational procedures, additional snapshots of aggregated peptides, alternative model assessments and further validations studies (PDF)
