# Supplementary material for: Beyond Tripeptides Two-Step Active Machine Learning for Very Large Data sets
Source: J Chem Theory Comput. 2021 Apr 27;17(5):3221–32. doi: 10.1021/acs.jctc.1c00159 (PMC8278388; doi:10.1021/acs.jctc.1c00159)
Supplement: Supplementary file 1 — ct1c00159_si_001.pdf [file ct1c00159_si_001.pdf]

Beyond tripeptides - two-step active machine learning for very large datasets

Alexander van Teijlingen, Tell Tuttle*

*tell.tuttle@strath.ac.uk

Supporting Information

Table of Contents

1. Experimental Procedures 3
   1.1 Wimley-White log P scale 3
   1.2 Mordred reduced feature set 3
   1.3 Mordred correlation matrices 5
   1.4 Hyperparameters 7
   1.5 ROC curves 9
   1.6 Model selection 11
   1.7 Initial training set 13
2. Results 16
   2.1 Validation of model accuracy 16
   2.2 Alternative methods 18
      2.2.1 Ranged sampling 18
      2.2.2 Active learning using only Judred descriptors 19
3. File Archive 21
   3.1 New computer programs 21
   3.2 Coarse-grained molecular dynamics data 23
   3.3 Active learning output 24
4. References 26

1. Experimental Procedures

1.1 Wimley-White log P scale

The log P for each peptide is calculate *via* the Wimley-White log P scale[1,2] in which the log P is derived from the octanol-to-water solvation free energies of individual amino acids (Equation S1)

Equation S1. Formula for determining log P of peptides proposed by W. Wimley & H. White[1,2] where N = number of amino acid residues in peptide.

$$log\ P = \sum_{i=1}^{N} \Delta G_{water-oct,i}$$

1.2 Mordred reduced feature set

Table S1. Mordred parameters used in this study

AATS0s	averaged moreau-broto autocorrelation of lag 0 weighted by intrinsic state
AATS2s	averaged moreau-broto autocorrelation of lag 2 weighted by intrinsic state
AATSC0are	averaged moreau-broto autocorrelation of lag 0 weighted by allred-rocow EN
AATSC0s	averaged and centered moreau-broto autocorrelation of lag 0 weighted by intrinsic state
AATSC0se	averaged and centered moreau-broto autocorrelation of lag 0 weighted by sanderson EN
AATSC1are	averaged and centered moreau-broto autocorrelation of lag 1 weighted by allred-rocow EN
AATSC1pe	averaged and centered moreau-broto autocorrelation of lag 1 weighted by pauling EN
AATSC1v	averaged and centered moreau-broto autocorrelation of lag 1 weighted by vdw volume
AATSC2s	averaged and centered moreau-broto autocorrelation of lag 2 weighted by intrinsic state
AATSC3d	averaged and centered moreau-broto autocorrelation of lag 3 weighted by sigma electrons
AMID_N	averaged molecular ID on N atoms
ATS4s	moreau-broto autocorrelation of lag 4 weighted by intrinsic state
ATSC1are	centered moreau-broto autocorrelation of lag 1 weighted by allred-rocow EN
ATSC1pe	centered moreau-broto autocorrelation of lag 1 weighted by pauling EN
ATSC1se	centered moreau-broto autocorrelation of lag 1 weighted by sanderson EN
ATSC4c	centered moreau-broto autocorrelation of lag 4 weighted by gasteiger charge
ATSC5c	centered moreau-broto autocorrelation of lag 5 weighted by gasteiger charge
ATSC5s	centered moreau-broto autocorrelation of lag 5 weighted by intrinsic state
AXp-0dv	0-ordered averaged Chi path weighted by valence electrons
BCUTm-1l	first lowest eigenvalue of Burden matrix weighted by mass
BIC1	1-ordered bonding information content
ETA_dEpsilon_B	ETA delta epsilon (type: B)
ETA_shape_p	ETA shape index (type: p)
ETA_shape_y	ETA shape index (type: Y)
FCSP3	the fraction of C atoms that are SP3 hybridized

GATS1are	geary coefficient of lag 1 weighted by allred-rocow EN
GATS1c	geary coefficient of lag 1 weighted by gasteiger charge
GATS1d	geary coefficient of lag 1 weighted by sigma electrons
GATS1se	geary coefficient of lag 1 weighted by sanderson EN
GATS2c	geary coefficient of lag 1 weighted by gasteiger charge
HybRatio	hybridization ratio
IC0	0-ordered neighborhood information content
IC1	1-ordered neighborhood information content
JGI2	2-ordered mean topological charge
Kier3	kappa shape index 3
MATS1v	moran coefficient of lag 1 weighted by vdw volume
MATS2p	moran coefficient of lag 2 weighted by pauling EN
MATS3v	moran coefficient of lag 3 weighted by vdw volume
NsNH2	number of sNH2
NsssN	number of sssN
piPC7	7-ordered pi-path count (log scale)
RotRatio	rotatable bonds ratio
SaasC	sum of aasC
SIC1	1-ordered structural information content
SlogP_VSA4	MOE logP VSA Descriptor 4 (0.00 <= x < 0.10)
SpMAD_A	SpMAD of adjacency matrix
Xc-5dv	5-ordered Chi cluster weighted by valence electrons

1.3 Mordred correlation matrices

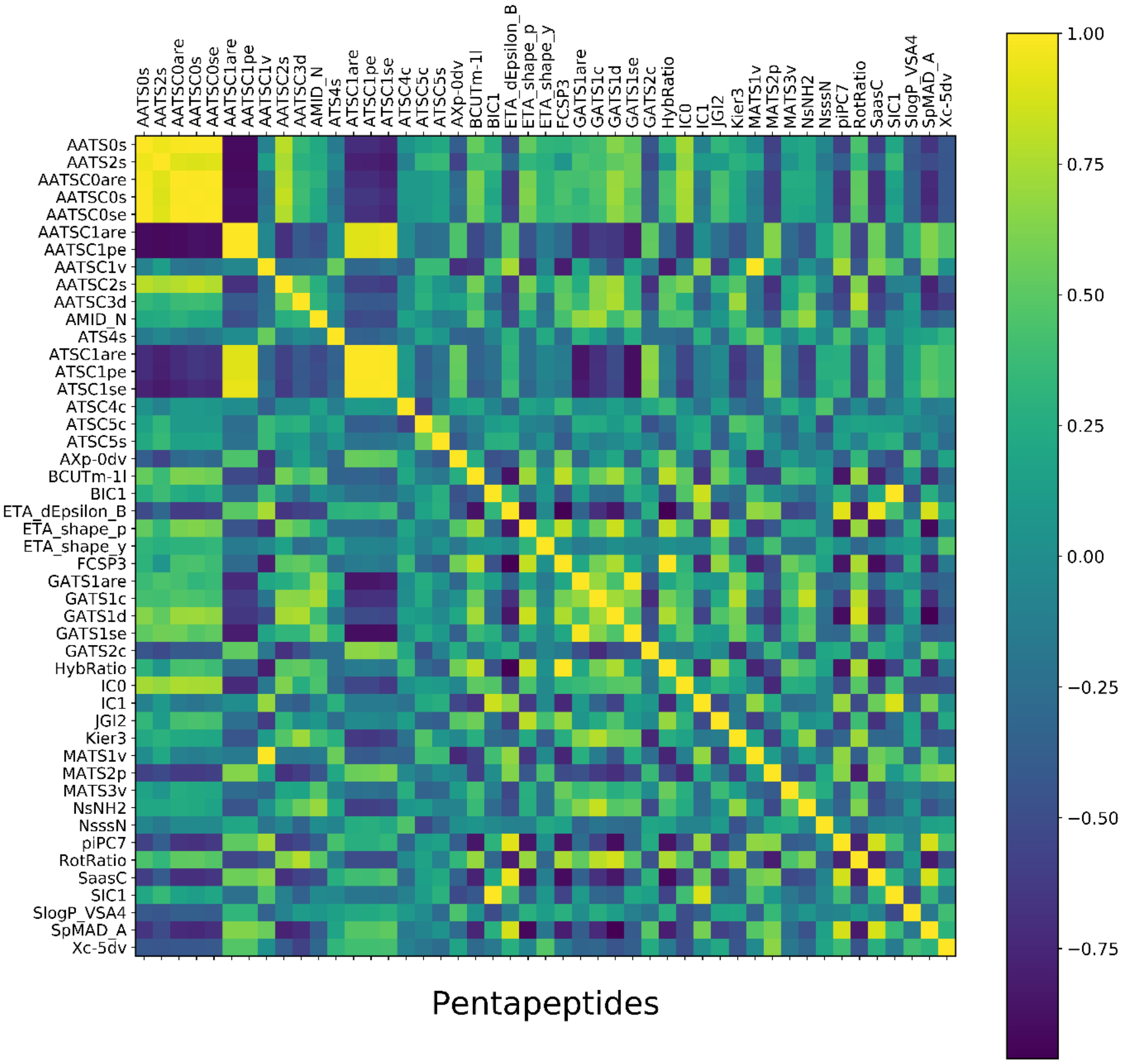

Figure S1. Correlation matrix of Mordred parameters for pentapeptides, some are highly correlated, but still vary between different peptide chain length datasets and therefore have been selected to remain.

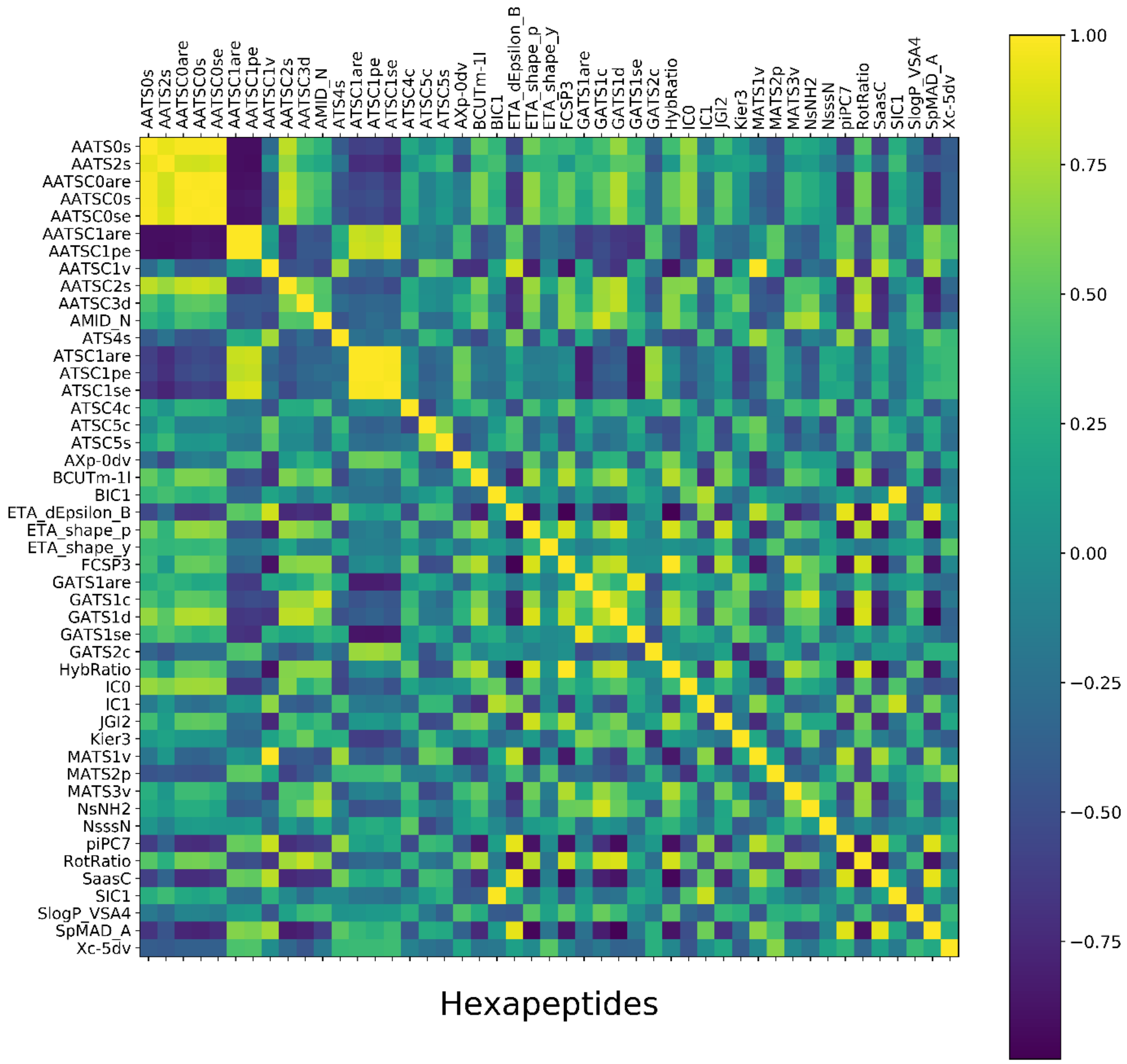

Figure S2. Correlation matrix of Mordred parameters for pentapeptides, some are highly correlated, but still vary between different peptide chain length datasets and therefore have been selected to remain.

1.4 Hyperparameters

Hyperparameters for each model were selected *via* 5-fold cross validation of the 80 % of the tripeptide dataset and validated against the remaining 20 %.

The training of the non-linear SVM using the radial basis function (SVM_{RBF}) for the kernel on Judred data had a C value of 100 and epsilon value of 0.1 with a scaled kernel coefficient. There was no limit on the number of iterations and the tolerance was 0.0001 (Table S2). In the case of training on Mordred data the C value was 30. The training of the elastic net model on Judred data used an alpha value of 0.001, L1 ratio of 0.1 with the number of iterations capped at 500 and a tolerance of 0.0001. The intercept was fit and the coefficients were updated by random selection on each iteration, these coefficients were allowed to be positive or negative. In the case of training on Mordred data the maximum number of iterations was capped at 1000. The training of the elastic net model on Judred data had a regularization parameter (C) of 100 and used the squared epsilon insensitive (L2) loss function with an epsilon vale of 0.1. The intercept was fit with an intercept scaling value of 0.1 and the number of iterations capped at 500 with a tolerance of 0.0001. In the case of training on Mordred data, C was 75 and the intercept scaling was 0.5. The training of the random forest model on Judred data used 300 estimators with no minimum impurity decrease and no early stopping threshold for tree growth. The minimum number of samples required to be a leaf node was 1 and the minimum weighted fraction of the sum of weights required to be a leaf node was 0. The quality measure of a split was measured as mean absolute error, boot strapping was disabled, and the maximum number of features considered when looking for best splits with the square root of the number of features. The use of out-of-bag samples to estimate r^2 on unknown data was disabled. These parameters were the same when training on Mordred data. The training of the ridge model on Judred data used and regularization strength of 0.1 with a regularized least squares algorithm was used to calculate the ridge coefficients. The intercept was fit with no limit of the number of iterations and a tolerance of 0.01. In the case of training on Mordred data a singular value decomposition to calculate the ridge coefficients and a tolerance of 0.01. The training of the GBR model on both the Judred and Mordred data used 150 estimators with a least squared regressor and a learning rate of 0.3 and Friedman mean square error criterion. The maximum depth of regression estimators was set to 3 and the minimum number of samples required to split a node set to 4. The training of the SGD model on Judred data used a squared epsilon insensitive loss function with a regularization strength of 0.0001 and an epsilon value of 0.01 with an L1 penalty. The learning rate was adaptive with an initial rate of 0.1 and an inverse scaling learning rate exponent of 0.25. The intercept was fit, and the maximum number of iterations capped at 3000 with a tolerance of 0.001. In the case of training on Mordred data, an L2 penalty was used with an epsilon insensitive loss function and an epsilon value of 0.1. The training of the MLP model on Judred data used a hidden layer with 100 nodes and rectified linear unit activation functions used on each. A constant learning rate of 0.001 was used with the maximum number of iterations capped at 300 and a tolerance of 0.0001. A Broyden–Fletcher–Goldfarb–Shanno (BFGS) solver was used for weight optimization with an L2 penalty of 0.0001. Batch gradient descent was used. In the case of training on Mordred data, mini-batch gradient descent was used with a batch size of 80, while the number of hidden layers was increased to 3. The training of the decision tree model on Judred data used a random splitter with no maximum depth, number of features or leaf nodes. The criterion optimised towards was the Friedman mean square error. The minimum impurity decrease for a node split was 0.02, the minimum number of samples required to be at a leaf node was 1 and the minimum number of samples required to split an internal node was 2.

Table S2. SVM_{RBF} hyperparameters

C	100
epsilon	0.1
gamma	scale
kernel	rbf
max_iter	-1
Tol	0.0001
Verbose	0

1.5 ROC curves

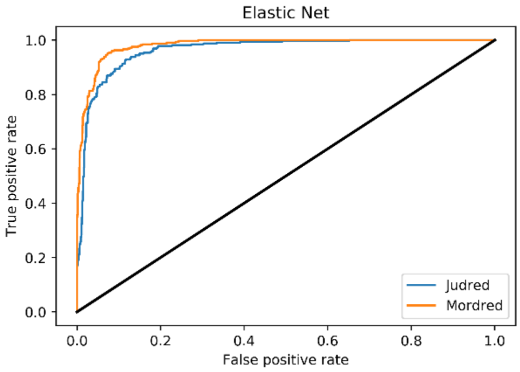

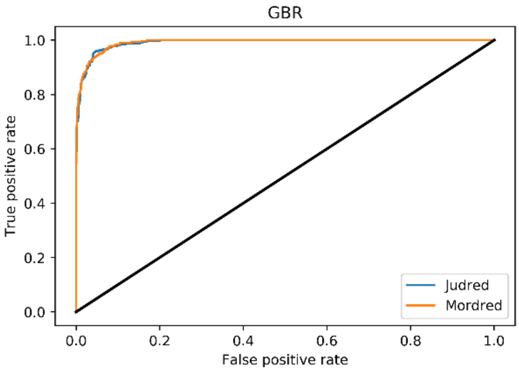

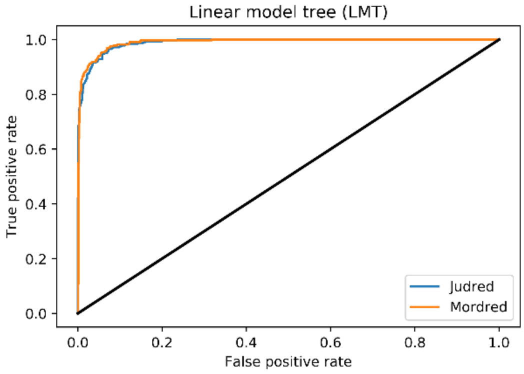

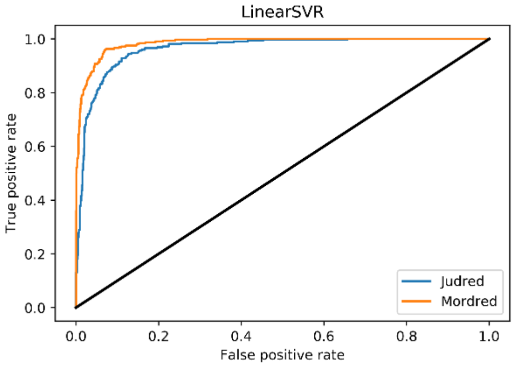

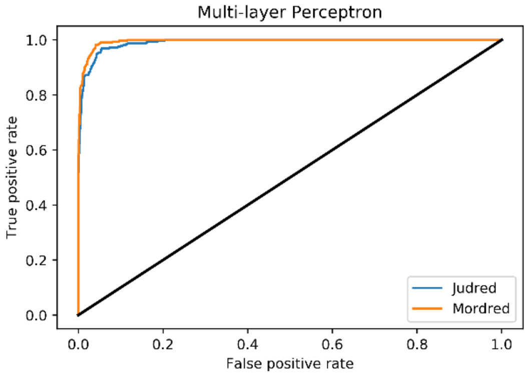

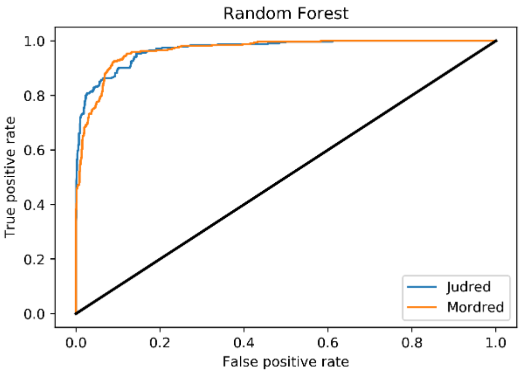

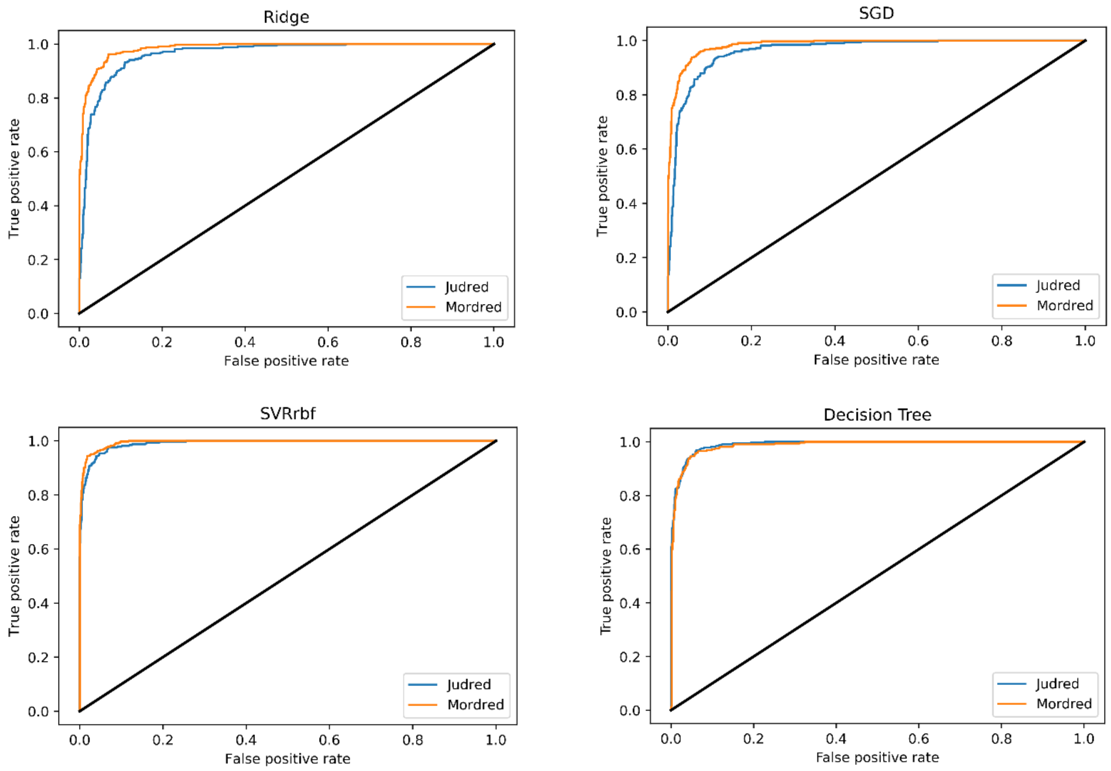

Figure S3. Receiver operating characteristic (ROC) curves for several different machine learning models attempting to distinguish between self-assembling and non-assembling peptides with the cut-off AP scoring being varied. In most cases, use of the Mordred dataset outperforms the Judred dataset, otherwise they perform evenly well.

1.6 Model selection

Table S3. Metrics for different machine learning algorithms using the Judred and Mordred datasets comparatively. A 2720-member test set (based on a 66:34 train:test split) was used to determine the best models according to five metrics, for MCC a cut-off of AP = 2.0 was used.

	Judred				
	MSE	MAE	R2	MCC	AUC
SVM_{RBF}	0.0162	0.0960	0.92	0.88	0.99
Linear SVM	0.0524	0.1874	0.76	0.67	0.96
Gradient boosting regressor	0.0155	0.0915	0.93	0.85	0.99
Elastic Net	0.0528	0.1877	0.75	0.7	0.96
Random Forest	0.0499	0.1728	0.77	0.75	0.97
Ridge	0.0520	0.1851	0.76	0.69	0.96
Multi-layer perceptron	0.0157	0.0932	0.93	0.88	0.99
Stochastic gradient descent	0.0520	0.1854	0.76	0.69	0.96
Decision Tree	0.0156	0.0906	0.93	0.87	0.99
	Mordred				
	MSE	MAE	R2	MCC	AUC
SVM_{RBF}	0.0078	0.0697	0.96	0.91	1.00
Linear SVM	0.0240	0.1251	0.89	0.83	0.99
Gradient boosting regressor	0.0099	0.0744	0.95	0.89	0.99
Elastic Net	0.0278	0.1349	0.87	0.79	0.98
Random Forest	0.0510	0.1737	0.76	0.73	0.97
Ridge	0.0240	0.1245	0.89	0.83	0.99
Multi-layer perceptron	0.0071	0.0636	0.97	0.9	0.99
Stochastic gradient descent	0.0244	0.1251	0.89	0.82	0.99
Decision Tree	0.0186	0.0969	0.91	0.87	0.99

Table S4. Metrics for different machine learning algorithms using the Judred and Mordred datasets comparatively. A 6400-member (80 %) training set to which the models had been hyperparameter optimised was used to measure model accuracy over 5-fold cross validation, for MCC a cut-off of AP = 2.0 was used.

	Judred				
	MSE	MAE	R2	MCC	AUC
SVM_{RBF}	0.0161 ± 0.0004	0.0962 ± 0.0017	0.925 ± 0.001	0.8825 ± 0.0132	0.9921 ± 0.001
Linear SVM	0.0558 ± 0.0019	0.1942 ± 0.0035	0.741 ± 0.008	0.6484 ± 0.0221	0.9615 ± 0.0029
Gradient boosting regressor	0.0157 ± 0.0005	0.0923 ± 0.0018	0.927 ± 0.002	0.8798 ± 0.0084	0.9918 ± 0.001
Elastic Net	0.0566 ± 0.0022	0.1942 ± 0.0038	0.738 ± 0.01	0.6663 ± 0.0154	0.9605 ± 0.0022
Random Forest	0.0486 ± 0.0023	0.1709 ± 0.0037	0.775 ± 0.011	0.7764 ± 0.0287	0.9725 ± 0.0038
Ridge	0.0556 ± 0.002	0.1923 ± 0.0036	0.742 ± 0.009	0.6655 ± 0.0276	0.9618 ± 0.0027
Multi-layer perceptron	0.0154 ± 0.0006	0.0927 ± 0.0021	0.929 ± 0.002	0.8909 ± 0.0062	0.992 ± 0.0014
Stochastic gradient descent	0.0556 ± 0.002	0.1925 ± 0.0036	0.742 ± 0.009	0.662 ± 0.0265	0.9617 ± 0.0027
Decision Tree	0.0169 ± 0.0004	0.0938 ± 0.0019	0.922 ± 0.002	0.8835 ± 0.0081	0.9913 ± 0.0014
	Mordred				
	MSE	MAE	R2	MCC	AUC
SVM_{RBF}	0.0079 ± 0.0003	0.0698 ± 0.0017	0.963 ± 0.001	0.8997 ± 0.012	0.9955 ± 0.0007
Linear SVM	0.0247 ± 0.0005	0.1259 ± 0.0016	0.885 ± 0.004	0.8333 ± 0.0144	0.9881 ± 0.0017
Gradient boosting regressor	0.0105 ± 0.0005	0.076 ± 0.0019	0.951 ± 0.002	0.8845 ± 0.0174	0.9931 ± 0.0012
Elastic Net	0.0286 ± 0.0007	0.1358 ± 0.0013	0.867 ± 0.006	0.7898 ± 0.0147	0.9839 ± 0.0026
Random Forest	0.0509 ± 0.0023	0.1748 ± 0.0034	0.764 ± 0.014	0.735 ± 0.0171	0.9707 ± 0.0024
Ridge	0.0246 ± 0.0005	0.1251 ± 0.0016	0.886 ± 0.004	0.8338 ± 0.013	0.9882 ± 0.0017
Multi-layer perceptron	0.0069 ± 0.0002	0.0627 ± 0.0009	0.968 ± 0.002	0.9045 ± 0.017	0.9955 ± 0.0012
Stochastic gradient descent	0.0252 ± 0.0005	0.1261 ± 0.0015	0.883 ± 0.004	0.8345 ± 0.0133	0.9882 ± 0.0019
Decision Tree	0.0191 ± 0.0009	0.0967 ± 0.0019	0.911 ± 0.003	0.8643 ± 0.0274	0.9881 ± 0.0023

1.7 Initial training set

In order to discern if the model was being biased heavily by this initial peptide we ran the model 20 times each time starting with a different sequence-uniform tripeptide, we found the maximum difference between any two mean AP values after 10 iterations to be 0.078, falling to 0.074 after 20 iterations, the plots have been visualised in the Supporting Information, Figure S4.

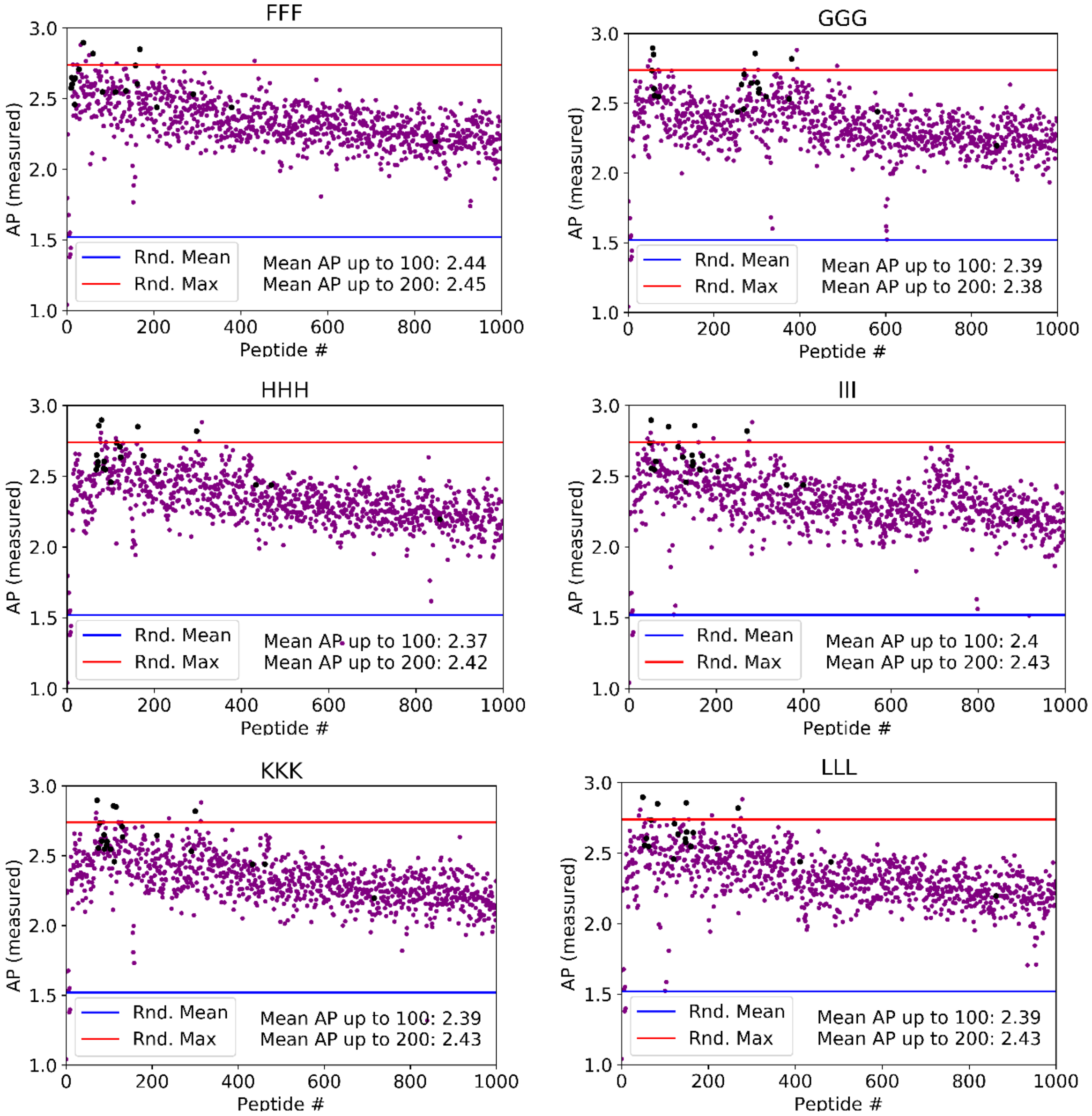

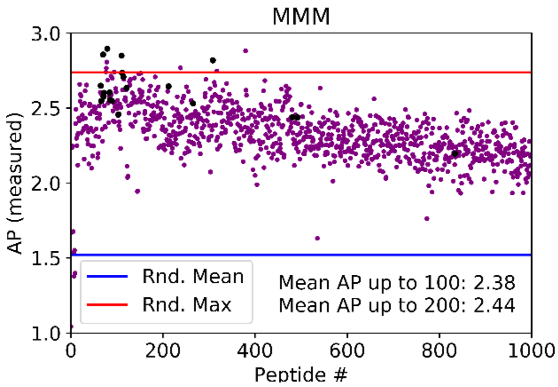
MMM
AP (measured)
Peptide #
Rnd. Mean
Rnd. Max
Mean AP up to 100: 2.38
Mean AP up to 200: 2.44

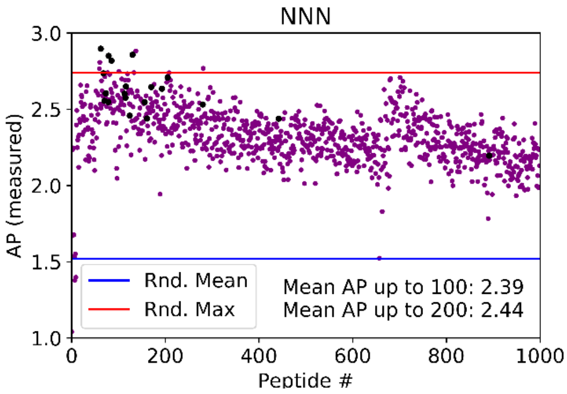
NNN
AP (measured)
Peptide #
Rnd. Mean
Rnd. Max
Mean AP up to 100: 2.39
Mean AP up to 200: 2.44

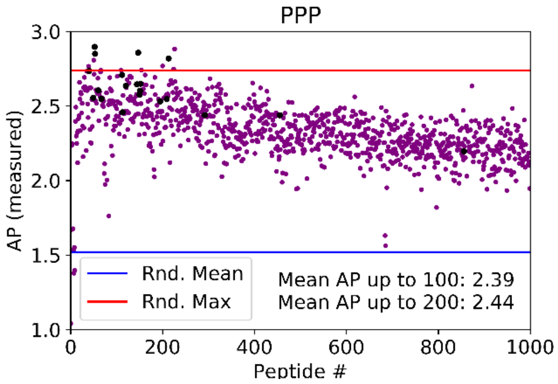
PPP
AP (measured)
Peptide #
Rnd. Mean
Rnd. Max
Mean AP up to 100: 2.39
Mean AP up to 200: 2.44

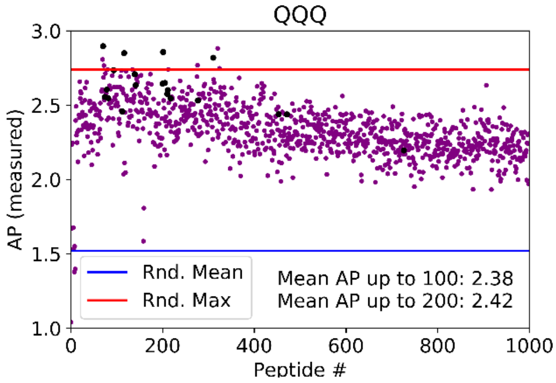
QQQ
AP (measured)
Peptide #
Rnd. Mean
Rnd. Max
Mean AP up to 100: 2.38
Mean AP up to 200: 2.42

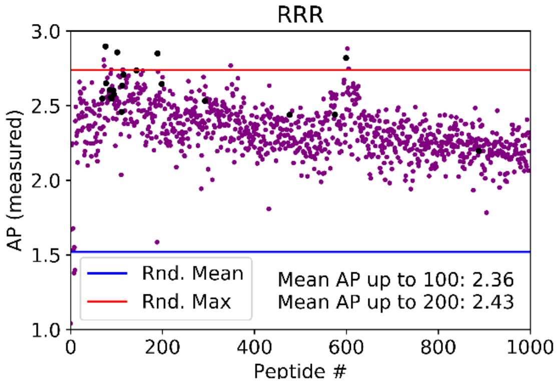
RRR
AP (measured)
Peptide #
Rnd. Mean
Rnd. Max
Mean AP up to 100: 2.36
Mean AP up to 200: 2.43

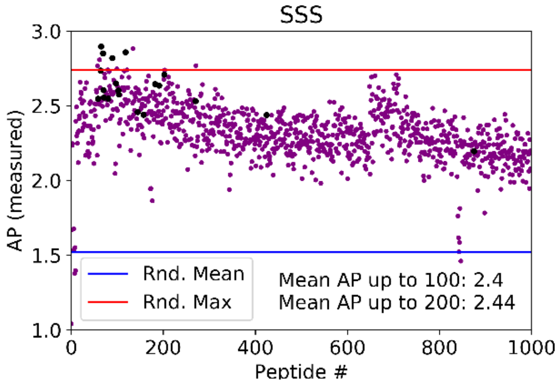
SSS
AP (measured)
Peptide #
Rnd. Mean
Rnd. Max
Mean AP up to 100: 2.4
Mean AP up to 200: 2.44

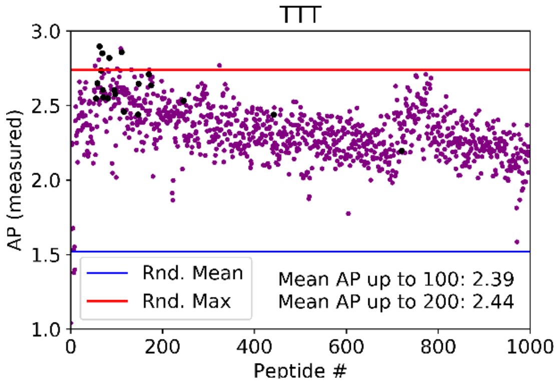
TTT
AP (measured)
Peptide #
Rnd. Mean
Rnd. Max
Mean AP up to 100: 2.39
Mean AP up to 200: 2.44

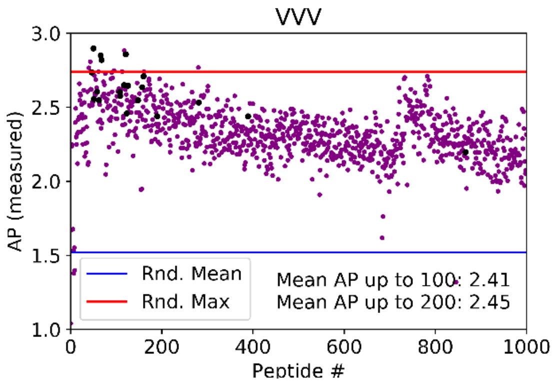
VVV
AP (measured)
Peptide #
Rnd. Mean
Rnd. Max
Mean AP up to 100: 2.41
Mean AP up to 200: 2.45

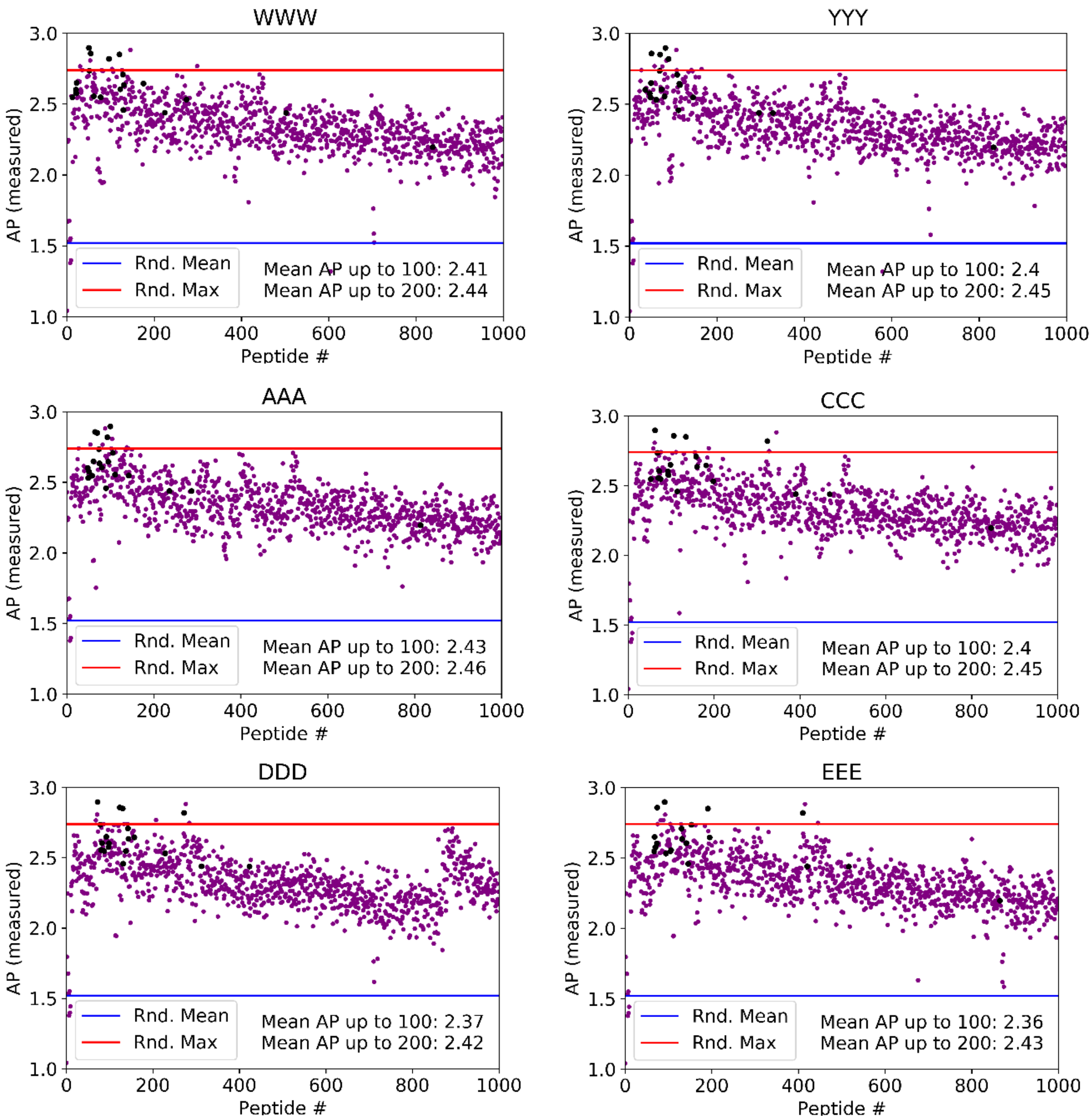

Figure S4. The initial training set used for active learning was the relevant polyalanine for the dataset in question. This figure shows that the model is not unfairly biased by the initial peptide choice, it merely functions to provide an initial step. Those reported by Frederix *et al.*[3] are shown in black.

2. Results

2.1 Validation of model accuracy

For each dataset 800 randomly selected peptides where selected and analysed *via* 5-fold cross-validation using the SVM_{RBF} machine learning model with optimized hyperparameters for both feature sets (Judred & Mordred), the Mordred predictions are found to be more accurate (Figure S5).

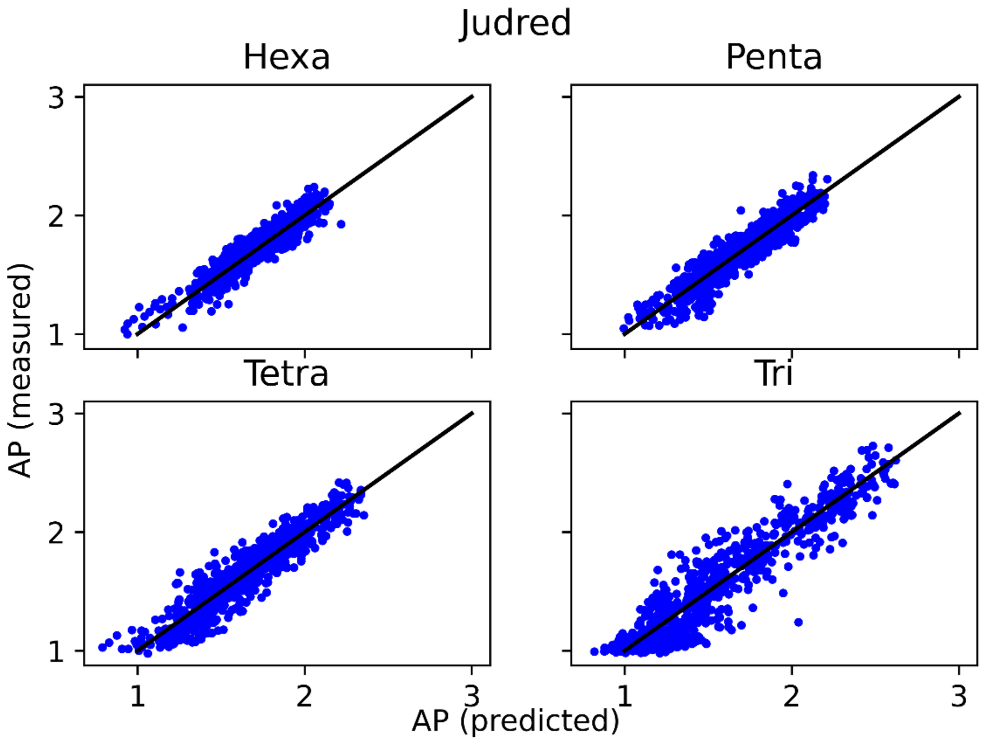

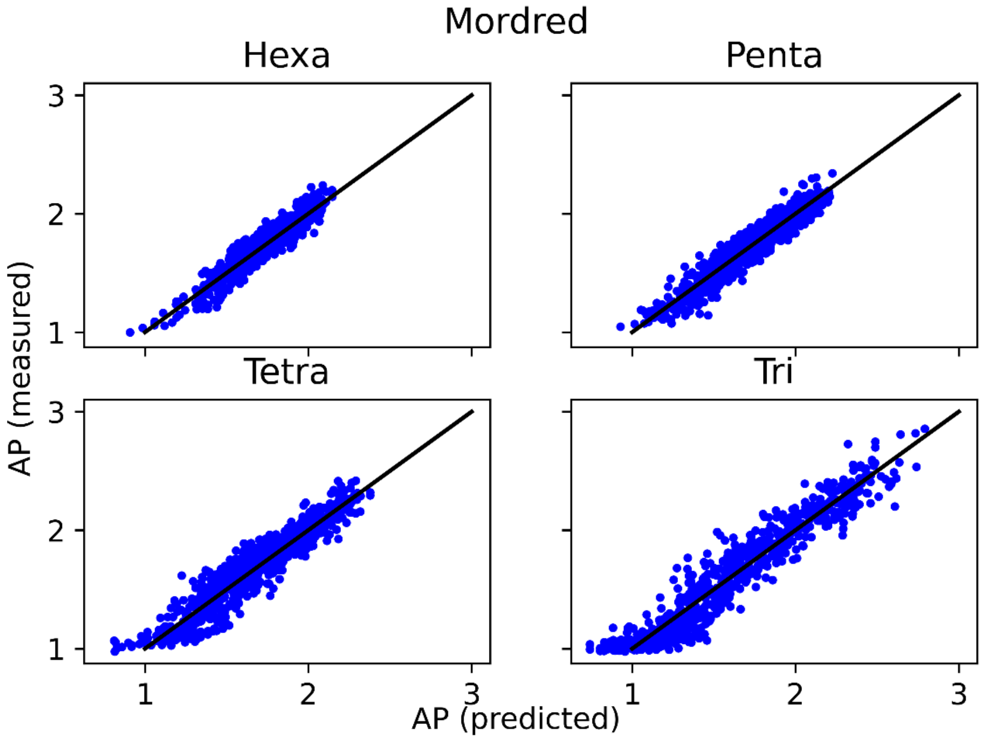

Figure S5. AP measured from CGMD simulation vs average AP predicted by our model *via* 5-fold cross validation of 800 randomly selected peptides from each dataset. The Mordred predictions are found to be more accurate with R^2 values between 0.87 – 0.91 and Judred R^2 values between 0.85 – 0.89.

2.2 Alternative methods

Other methods of sampling such as each iteration consists of the best (highest AP) tripeptide and 9 randomly chosen weighted tripeptides (weight = AP) are analysed to compare to our method, we find that they do not produce as impressive results.

2.2.1 Ranged sampling

Instead of submitting the top 10 AP predictions for CGMD simulations we tested Range Sampling by submitting the highest AP tripeptide and 9 randomly chosen weighted tripeptides where the weight was equal to the AP score, this method found 19/20 of the top tripeptides reported by Tuttle *et al*.[3] (compared to 20/20 for the top 10 AP predictions) with the top predictions being spread into later rather than earlier iterations of the model (Figure S6). The mean AP score up to 100 peptides (10 iterations) is 2.43 for the Top 10 Learning model and 2.22 for the Range Sampling model. By the 200th peptide (20th iteration) the difference in the mean AP score is essentially retained with the Top 10 model returning a mean AP score of 2.46 and for the Range Sampling model 2.25 for the tripeptide series.

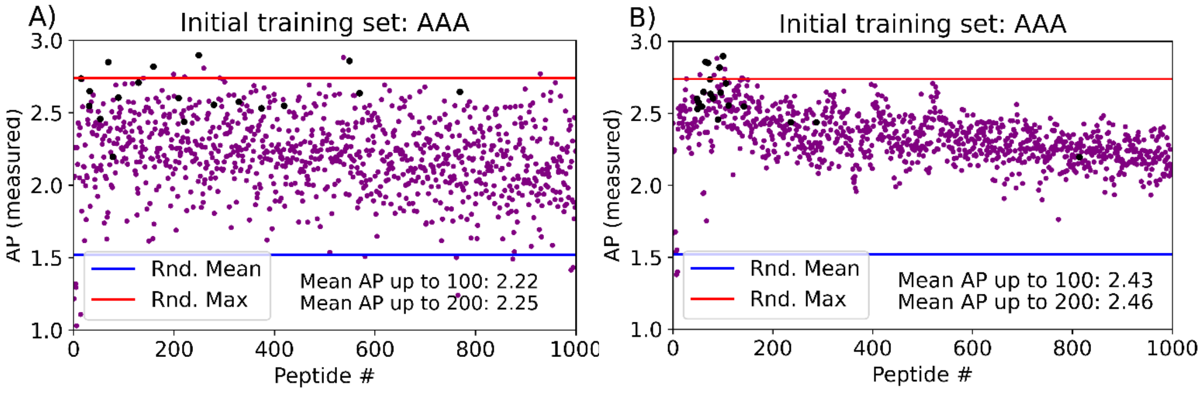

Figure S6. Tripeptides active learning where A) each iteration consists of the best (highest AP) tripeptide and 9 randomly chosen weighted tripeptides (weight = AP) and B) each iteration consists of the 10 best predicted peptides. Black dots show the top tripeptides reported by Frederix *et al*.[3]. Inset is the mean AP score of peptides found after 100 and 200 peptides (10 & 20 iterations).

2.2.2 Active learning using only Judred descriptors

We also compared using a Judred only based active learning method without the second step of predicting based on Mordred descriptors. The results were compared for tetrapeptides with no restrictions on log P and the restrictions of max = 0 and max = -4, the results were more sporadic and tended to learn slowly than the two-step model (Figure S7).

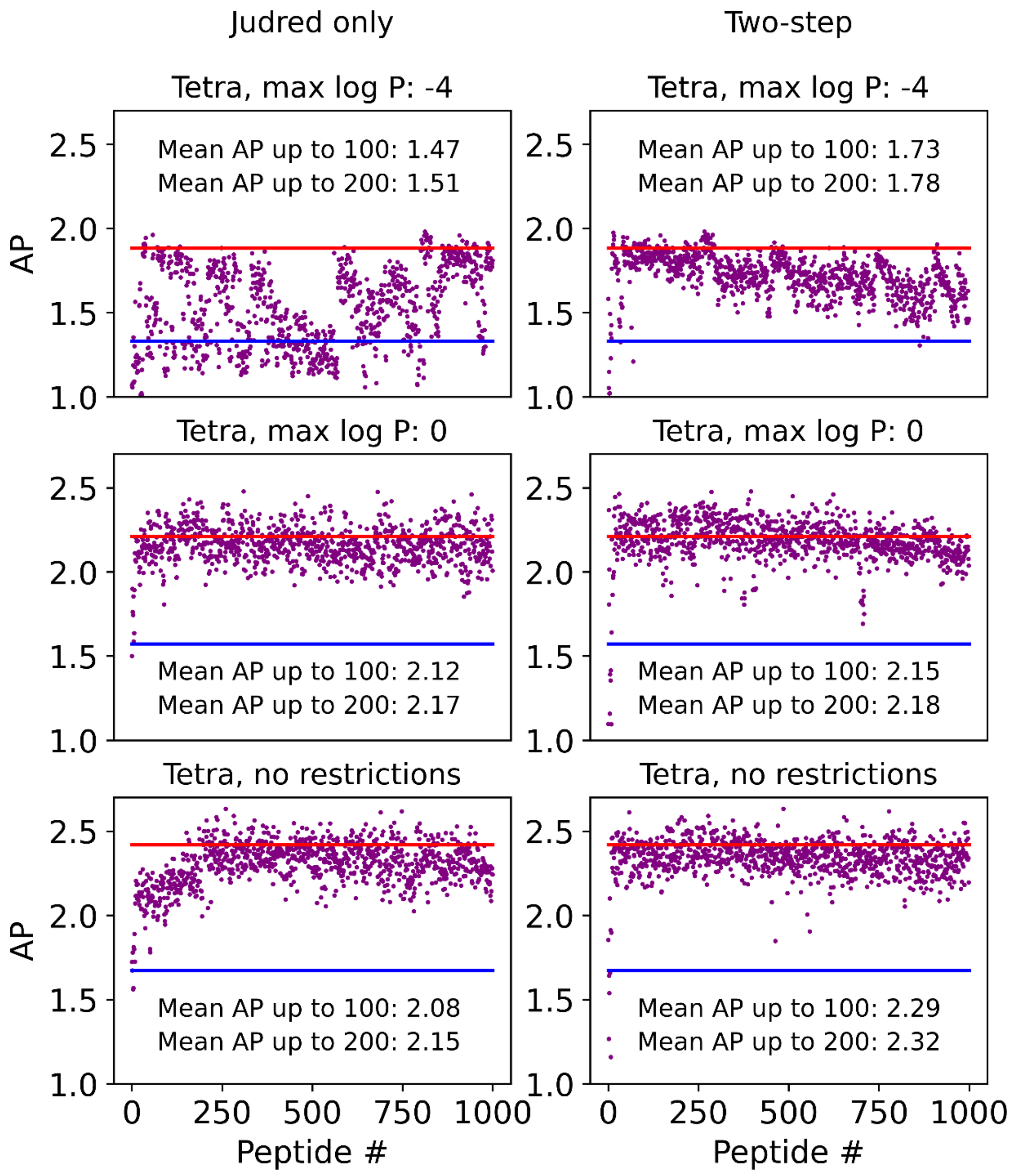

Figure S7. The dual-resolution algorithm is compared to a Judred-only version of the algorithm for tetrapeptides with no restrictions on the dataset and with varying restrictions on the dataset. The results show that the Judred-only model takes longer to learn the trends for predicting a high AP score and returns more erratic results when tasks with the harder job of trying to predict aggregating/self-assembling soluble peptides. The red and blue lines represent the maximum and mean values from the random dataset respectively. Inset is the mean AP score of peptides found after 100 and 200 peptides (10 & 20 iterations).

3. File Archive

3.1 New computer programs

All data underpinning this publication are openly available from the University of Strathclyde KnowledgeBase at https://doi.org/10.15129/46bc0a77-6c7f-4a13-8b07-33a50d0129b0

The authors have included the core and auxiliary source code of the active learning process, the files, their descriptions and paths are listed in Table S5.

Table S5. Python, Julia and Bash scripts we used to implement the active learning method described.

Entry	Description	Language	File path
1	Generate of Judred datasets for a given range of peptides within a set length of amino acids	Julia	Scripts.zip/ Judred/Judred.jl
2	Subroutine used by the Judred program to convert between three-letter peptide sequences and one-letter peptide sequences	Julia	Scripts.zip/ Judred/peptideutils.jl
3	Main two-step active learning routine	Python	Scripts.zip/ ActiveLearnMordred/ ActiveLearnMordred2.py
4	Main Judred data only active learning routine	Python	Scripts.zip/ ActiveLearnMordred / ActiveLearnMordred2 _JudredOnly.py
5	Subroutine used by ActiveLearnMordred2.py for loading Judred data and pre-screening datasets.	Python	Scripts.zip/ ActiveLearnMordred / prescreen.py
6	Subroutine used by ActiveLearnMordred2.py for managing the submitting of CGMD simulations and calculating AP scores.	Python	Scripts.zip/ active_learning/ __init__.py
7	Subroutines for querying the Slurm job queue.	Python	Scripts.zip/ hpctools/ __init__.py
8	Subroutine used by the python programs to convert between three-letter peptide sequences and one-letter peptide sequences	Python	Scripts.zip/ peptideutils/ __init__.py
9	Generation of Gromacs input files and submission of CGMD simulations	Python	Scripts.zip/ APMD/ apmd.py
10	Loads required modules on a Slurm based high performance computer (HPC) and passes arguments to apmd.py	Bash	Scripts.zip/ APMD/ apmd.sh
11	Program for optimizing hyperparameters for each machine learning algorithms for	Python	Scripts.zip/ ActiveLearnMordred/ BestScreenML.py

	prediction accuracy against the tripeptides dataset		
12	Program for querying the status of CGMD jobs and restarting those that have failed or timed out.	Python	Scripts.zip/<br>APMD/<br>SimChecker.py

3.2 Coarse-grained molecular dynamics data

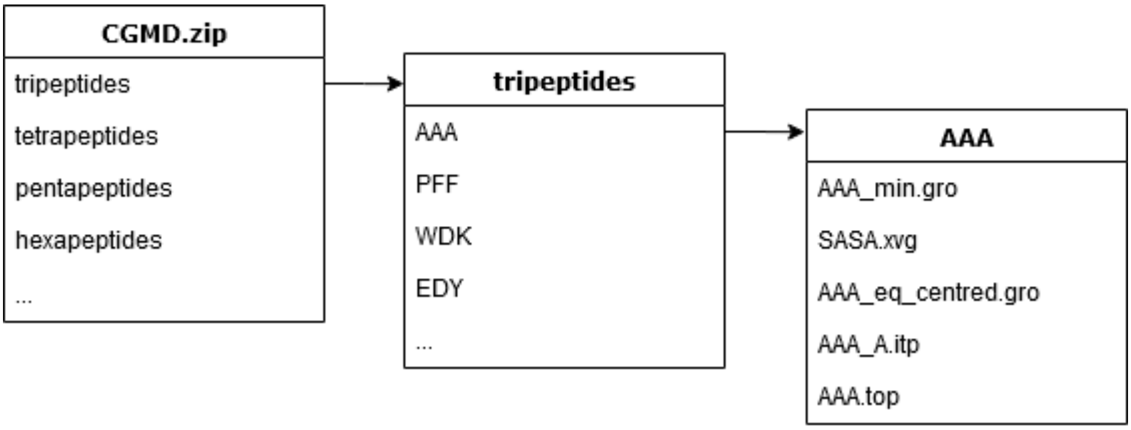

Figure S8. Directory and file structures of coarse-grained molecule dynamics (CGMD) data.

Figure S8 shows the file structure of the CGMD zip file, which contains a zip file for each active learning process reported. Each zip file contains folders for each peptide which in turn contain the following (where * represents a single letter peptide code):

- *_min.gro: first frame, from which $SASA_{initial}$ is calculated
- *_eq_centred.gro – final frame, from which $SASA_{final}$ is calculated
- SASA.xvg – SASA at each frame of the trajectory (not included due to size)
- *_A.itp – MARTINI topology of peptide
- *.top – topology of system

The content of the CGMD if given in Table S6.

Table S6. CGMD input parameters, output files and calculated results.

Entry	Description	Data type	File path
1	AP scores measured for 59,936 peptides from di- to octa-included all those reported herein, values may differ from other studies that did not use the MARTINI version 2.2 force field.	CSV (space separated)	AP_50ns.txt
2	Minimization parameters	Gromacs input	CGMD.zip/water_min.mdp
3	Equilibration parameters	Gromacs input	CGMD.zip/water_eq_50ns.mdp
4	MARTINI forcefield version 2.2	Gromacs forcefield	CGMD.zip/martini_v2.2.itp
5	MARTINI ions topology	Gromacs topology	CGMD.zip/martini_v2.0_ions.itp
6	Relevant gromacs output for each tripeptide reported by our active learning method.	Gromacs output	CGMD.zip/tripeptides
7	Relevant gromacs output for each tetrapeptide reported by our active learning method.	Gromacs output	CGMD.zip/tertapeptides
8	Relevant gromacs output for each pentapeptide reported by our active learning method.	Gromacs output	CGMD.zip/pentapeptides
9	Relevant gromacs output for each hexapeptide reported by our active learning method.	Gromacs output	CGMD.zip/hexapeptides

3.3 Active learning output

Table S7. Output logs from the active learning process, break lines are included between each iteration of 10 peptides and the initial polyalanine is not logged.

Entry	Description	File path
1	Active learning results using the tripeptides dataset without log P restrictions	logs.zip/NewTripeptides_LOGP1000_N=1_SVRrbf.txt
2	Active learning results using the tripeptides dataset restricted to only those peptides with a log P below 0	logs.zip/NewTripeptides_LOGP0_N=1_SVRrbf.txt
3	Active learning results using the tripeptides dataset restricted to only those peptides with a log P below -3	logs.zip/NewTripeptides_LOGP-3_N=1_SVRrbf.txt
4	Active learning results using the tetrapeptides dataset without log P restrictions	logs.zip/NewTetrapeptides_LOGP1000_N=1_SVRrbf.txt
5	Active learning results using the tetrapeptides dataset	logs.zip/NewTetrapeptides_LOGP0_N=1_SVRrbf.txt

	restricted to only those peptides with a log P below 0	
6	Active learning results using the tetrapeptides dataset restricted to only those peptides with a log P below -4	logs.zip/NewTetrapeptides_LOGP-4_N=1_SVRrbf.txt
7	Active learning results using the pentapeptides dataset without log P restrictions	logs.zip/NewPentapeptides_LOGP1000_N=1_SVRrbf.txt
8	Active learning results using the pentapeptides dataset restricted to only those peptides with a log P below 0	logs.zip/NewPentapeptides_LOGP0_N=1_SVRrbf.txt
9	Active learning results using the pentapeptides dataset restricted to only those peptides with a log P below -4	logs.zip/NewPentapeptides_LOGP-4_N=1_SVRrbf.txt
10	Active learning results using the hexapeptides dataset without log P restrictions	logs.zip/NewHexapeptides_LOGP1000_N=1_SVRrbf.txt
11	Active learning results using the hexapeptides dataset restricted to only those peptides with a log P below 0	logs.zip/NewHexapeptides_LOGP0_N=1_SVRrbf.txt
12	Active learning results using the pentapeptides dataset restricted to only those peptides with a log P below -4	logs.zip/NewHexapeptides_LOGP-4_N=1_SVRrbf.txt

4. References

[1] W.C. Wimley, T.P. Creamer, S.H. White, Solvation energies of amino acid side chains and backbone in a family of host - Guest pentapeptides, Biochemistry. 35 (1996) 5109–5124. https://doi.org/10.1021/bi9600153.

[2] S.H. White, W.C. Wimley, Hydrophobic interactions of peptides with membrane interfaces, Biochim. Biophys. Acta - Rev. Biomembr. 1376 (1998) 339–352. https://doi.org/10.1016/S0304-4157(98)00021-5.

[3] P.W.J.M.J.M. Frederix, G.G. Scott, Y.M. Abul-Haija, D. Kalafatovic, C.G. Pappas, N. Javid, N.T. Hunt, R. V. Ulijn, T. Tuttle, Exploring the sequence space for (tri-)peptide self-assembly to design and discover new hydrogels, Nat. Chem. 7 (2015) 30–37. https://doi.org/10.1038/nchem.2122.
